# Fermentation capacity of the gut microbiota influences exercise motivation and neuroendocrine integration

**DOI:** 10.1128/msystems.00876-26

**Published:** 2026-08-19

**Authors:** Noah Thomas Hutchinson, Christian A. Maino-Vieytes, Cristian Valls, Jacob Allen, Lauretta A. Rund, Rodney W. Johnson, Jeffrey A. Woods

**Affiliations:** 1 Division of Nutritional Sciences, University of Illinois at Urbana-Champaign (UIUC) 14589, Urbana, Illinois, USA; 2 Department of Biomedical Engineering, University of Michigan 1259, Ann Arbor, Michigan, USA; 3 DC Health 11197 , Washington, DC, USA; 4 Department of Pediatrics, University of Michigan 1259, Ann Arbor, Michigan, USA; 5 Department of Health and Kinesiology, University of Illinois at Urbana-Champaign 14589, Urbana, Illinois, USA; 6 Department of Animal Sciences, University of Illinois at Urbana-Champaign 14589, Urbana, Illinois, USA; The University of Texas at Dallas, Richardson, Texas, USA

**Keywords:** gut-brain axis, short-chain fatty acids, exercise motivation, functional metagenomics, HPA axis, voluntary wheel running, neuroendocrine integration, prebiotics

## Abstract

Physical inactivity contributes substantially to global disease burden, yet the physiological mechanisms underlying exercise motivation remain poorly understood. The gut-brain axis presents a potentially modifiable target for behavioral intervention. Emerging evidence demonstrates that the gut microbiota influences motivated behaviors, but the specific metabolic functions and physiological mechanisms mediating these effects remain poorly defined. Here, we demonstrate that the predicted fermentation capacity of the gut microbiota influences voluntary wheel running (VWR) acquisition and neuroendocrine integration during exercise in C57BL/6J mice. Antibiotic-induced microbiome depletion reduced VWR acquisition, while shifting predicted function toward aerobic respiration and away from anaerobic fermentation. Supplementation with short-chain fatty acids, the primary fermentative products, restored normal VWR activity in microbiome-depleted mice. Conversely, 4-week dietary pretreatment with 2.5% prebiotic fiber (inulin) increased predicted fermentative capacity of the microbiota and VWR activity above baseline levels. Microbiome manipulation produced bidirectional dysregulation of corticosterone responses to exercise: acute antibiotic depletion increased post-exercise concentrations, while germ-free development decreased them, despite elevated striatal catecholamines. This exercise-specific uncoupling reveals microbiome-dependent integration of metabolic demand signals in the coordination of sympathetic and hypothalamic-pituitary-adrenal axis responses. Furthermore, the inulin-induced enhancement in VWR activity was associated with increased striatal histamine concentrations following exercise, suggesting additional mechanisms of neuromodulation. These findings demonstrate that the fermentative capacity of the gut microbiota influences exercise motivation and neuroendocrine regulation, providing novel insights into dietary interventions targeting physical activity.

**IMPORTANCE:**

Physical inactivity is a leading cause of global morbidity and mortality, and our lack of understanding of the biological forces driving motivation to exercise limits our ability to develop interventions that enhance engagement. Using a rodent model of voluntary exercise along with microbiota depletion and metabolite replacement, we uncovered that the gut microbiota and its capacity to ferment dietary components into short-chain fatty acids drive exercise habit acquisition and help facilitate coordination between neurochemical signals and systemic stress hormones during exercise. Additionally, microbiome depletion “uncoupled” these systems, resulting in dysregulated stress responses during forced exercise. Finally, we showed that enhancement of microbiota fermentation capacity via dietary addition of prebiotic fiber was able to increase exercise engagement while also enhancing concentrations of histamine, a neuromodulator that potentiates locomotor activity, in the striatum. These findings suggest that the gut microbiome is a modifiable target for behavior change that facilitates integration of metabolic demand in neuroendocrine activity. Collectively, this work provides a mechanistic foundation to support the use of dietary interventions in sedentary populations to start exercise habits.

## INTRODUCTION

The physical inactivity of over 50% of adults worldwide is responsible for an estimated $53.8 billion in healthcare expenditure and roughly 9% of premature mortality, and current interventions fail to induce motivation to exercise (1–6). Recent research into the gut-brain axis indicates that microbiota metabolic function influences motivated behaviors through modulation of neuroendocrine and neurochemical activity (7, 8). Primarily through anaerobic fermentation, gut microbes generate a plethora of bioactive metabolites, the most prominent being short-chain fatty acids (SCFAs) that influence central nervous system (CNS) function through a variety of enteroendocrine and neural pathways (9). Fermentation capacity, the microbiome’s ability to incompletely oxidize dietary substrates into bioactive molecules, represents a measurable and modifiable metabolic function via behavioral or dietary means (10, 11). Dysbiotic states induced by antibiotic treatment (ABX) impair fermentation through depletion of obligate anaerobes, while consumption of prebiotic fiber enhances it. Given the suboptimal fiber intake among western populations, dietary prebiotic fiber interventions represent an unexplored approach to increase physical activity through potentiating microbiota fermentation capacity.

Rodent voluntary wheel running (VWR) patterns mirror reward-seeking behaviors, with an early “acquisition” phase dependent on neural reward circuits (12, 13), followed by a “maintenance” phase of habitual control (14). We(8)and others (10) have also shown that prior ABX and germ-free status diminish daily VWR volume during the acquisition phase, establishing that an intact microbiota is required for optimal acquisition of this motivated behavior.

It remains unclear which microbial functions and physiological signaling pathways mediate this relationship. First, the metabolic functions through which the microbiota exerts its influence remain largely uncharacterized. Second, while it is known that SCFAs initiate vagal afferent signaling through free fatty acid receptors (FFARs) on enteroendocrine cells to influence hypothalamic-pituitary-adrenal (HPA) axis activity (15), it is unknown whether microbiome perturbation affects coordination of sympathetic and HPA axis responses during exercise. Since the HPA axis engages in bidirectional regulation with energy substrates and arousal signals through glucocorticoid release, understanding microbiome influence on HPA activity during exercise could provide valuable insights into sustaining motivated behavior (16, 17). Furthermore, it remains unexplored whether dietary interventions that alter microbiota composition and enhance fermentation capacity can be leveraged to potentiate exercise motivation through this pathway.

To address these knowledge gaps, we examined how interventions targeting fermentative output, such as antibiotic depletion, SCFA supplementation, and prebiotic enrichment, influence VWR acquisition and associated neuroendocrine responses. We hypothesized that the fermentation capacity of the microbiota would causally modulate exercise motivation during the acquisition period, such that its depletion would impair and its enhancement would augment VWR activity, and that these behavioral phenotypes would be accompanied by concurrent changes in gut-brain neuroendocrine signaling relevant to stress and reward pathways that regulate exercise behavior. Using bioinformatic tools for functional metagenomic prediction alongside behavioral and physiological phenotyping, we demonstrate that predicted fermentative capacity of the microbiota is associated with exercise motivation. Finally, we demonstrate initial evidence of the microbiota’s role in neuroendocrine integration of metabolic signals during exercise.

## MATERIALS AND METHODS

### Animals and treatments

Ninety 6-week-old C57BL/6J male mice were ordered from Jackson Laboratories (Bar Harbor, ME), and twenty 8-week-old male C57BL/6J germ-free (GF) mice were received from the University of Illinois at Urbana-Champaign in-house gnotobiotic colony, which was also derived from Jackson Laboratories. After a 2-week acclimation period, mice were randomized to treatment groups based on body weight, and horizontal telemetered running wheels (Med Associates, Fairfax, VT) were placed in their cages. For the first experiment, treatments (ABX vs unaltered microbiome control (CON)) began either 3 days prior or 3 weeks into the 6-week VWR period to target acquisition and maintenance phases, respectively. For the remainder of the experiments, treatments consisted of CON, ABX, starting 3 days prior to and throughout the VWR trial period, aside from the 2.5% inulin and 1% cellulose-enhanced AIN3M diet (Envigo), which was started 4 weeks prior to VWR. ABX consisted of vancomycin hydrochloride (4.5 mg·kg^−1^·day^−1^), kanamycin sulfate (40 mg·kg^−1^·day^−1^), ampicillin (33 mg·kg^−1^·day^−1^), and metronidazole (21.5 mg·kg^−1^·day^−1^) delivered in the drinking water. Similar broad-spectrum mixtures have been shown to significantly deplete microbes, especially within the gut, and not cause overt sickness, weight loss, or systemic inflammation (8, 18–20). Cecum weight was used as a proxy for the success of antibiotic-induced microbiota depletion, as this is commonly observed in microbiota depletion models (Fig. S1; 18–20). For all treatment groups, cecum weight at sacrifice was recorded and was used to display successful depletion or absence of the native microbiota, as this effect has been established in numerous studies. Supplemental SCFA treatment followed a previously utilized cocktail including 67.5 mM sodium acetate, 25.9 mM sodium propionate, and 40 mM sodium butyrate administered in drinking water. This mixture was chosen as it has previously induced benefits in the CNS, promoting healthy maturation of microglia (21), and attenuation of stress-induced reduction in reward-seeking behavior (22). The 2.5% inulin diet was chosen as it has been shown to induce increases in cecal butyrate concentrations and exert beneficial effects on microglial transcriptomes (23). Only male mice were used in this study. This decision was informed by observations from related work in our laboratory using this antibiotic paradigm, in which effects on voluntary wheel running appeared more pronounced in males, although this comparison was not formally reported. We recognize this as a limitation, given known sex differences in gut microbiota composition, stress-axis reactivity, and exercise behavior more broadly, and identify direct investigation of sex as a biological variable as a priority for future studies in this line of research (see Discussion).

### Sample size and power analysis

No prospective a priori power calculation was performed prior to these studies. Sample sizes for the antibiotic cohort were instead informed by a prior study in our laboratory using the same antibiotic cocktail and voluntary wheel running paradigm (8), which detected a significant effect of treatment on running behavior at *n* = 10/group; subsequent expansion of the factorial ABX × SCFA design to *n* ≥ 14/cell reflected the reduced per-comparison degrees of freedom inherent to a 2 × 2 design. Sample size for the inulin cohort followed similar lab convention for this paradigm.

To characterize the magnitude of treatment effects on voluntary wheel running, the primary behavioral outcome, we report partial eta-squared (η²p) and Cohen’s *f* for the treatment-related fixed effects from each cohort’s linear mixed-effects model (treatment × day interaction, random intercept per animal, as described above). For the two-arm antibiotic cohort (ABX vs CON, *n* = 9/group), the treatment effect yielded η²p = 0.253 (Cohen’s *f* = 0.583). For the 2 × 2 antibiotic-by-SCFA cohort (*n* ≥ 14/cell), the ABX main effect yielded η²p = 0.121 (*f* = 0.370), and the ABX × SCFA interaction yielded η²p = 0.033 (*f* = 0.186). For the inulin cohort (*n* = 10/group), the diet main effect yielded η²p = 0.132 (*f* = 0.391).

We additionally estimated retrospective statistical power for these comparisons via parametric bootstrap simulation (500 simulations per cohort; simr package, Green and MacLeod 2016), computed as the proportion of simulated data sets in which the full treatment model significantly outperformed a day-only baseline model (likelihood ratio test, *P* < 0.05; Table S1). Because retrospective power calculated from an observed effect size is mathematically related to the achieved significance of that effect, these estimates characterize detected effect magnitude rather than provide independent confirmation that sample sizes were adequately powered a priori. As a sensitivity check, we also computed power using a classical repeated-measures analysis of variance (ANOVA) framework with Greenhouse-Geisser correction for sphericity violations (empirical ε ≈ 0.25 across cohorts, consistent with the strong autocorrelation typical of daily activity data); this more conservative approach yielded substantially lower power estimates (range: 0.08–0.29; Table S1), reflecting the lower statistical efficiency of classical ANOVA relative to the mixed-effects framework used for our primary analyses.

### 16S rRNA sequencing and taxonomic analyses

The fecal samples used for microbiome analyses in this study were collected from two independent and separate cohorts. The antibiotic-treated cohort, which is previously published under different analysis parameters (8), received the described four-antibiotic cocktail or water throughout 6 weeks of VWR. Fecal samples collected after VWR completion were analyzed to examine the relationship between microbiome depletion and the reduced VWR engagement observed in that study. Post-VWR samples were used, since pre-samples were collected prior to antibiotic treatment, and it was deemed that antibiotic treatment would be a much more potent stimulus than VWR, thus still allowing us to investigate the microbiome characteristics that drove reduced running behavior. This was confirmed by beta-diversity and permutational multivariate analysis of variance (PERMANOVA) analyses at the pre-treatment timepoint, which found no difference in community structure between mice subsequently assigned to antibiotic or control treatment, while post-antibiotic samples showed clear separation (see Results). Thus, post-treatment samples were deemed to be the relevant comparison for downstream behavioral and neurochemical analyses. The inulin-supplemented cohort used the same diet described here, but mice were treated for 8 weeks, as described (22). Mice received either the traditional chow diet or the described 2.5% inulin-enhanced diet. Given the more subtle and targeted nature of inulin supplementation, analyses of that cohort were conducted longitudinally.

For both cohorts, bacterial DNA was extracted using the DNeasy PowerSoil Pro Kit (Qiagen) and sequenced either the V3–V4 region (inulin study) or the V4 region (ABX study) of the 16S rRNA gene. Sequencing was performed on an Illumina MiSeq platform (2 × 250 bp paired-end reads). Raw sequences were processed using DADA2 v1.28 (24) to generate amplicon sequence variants (ASVs) with default quality-filtering parameters.

Taxonomic assignments were made using the Silva v138 reference database (25). The antibiotic cohort was analyzed cross sectionally, with relative abundance comparisons between the ABX and control mice at the post-intervention timepoint, after ABX and VWR. ASV count tables were converted to relative abundances (proportions) by dividing each ASV count by the total read count per sample. Statistical comparisons were performed using Wilcoxon rank-sum tests for individual taxa, with Benjamini-Hochberg false discovery rate (FDR) correction for multiple testing. Taxa present in ≥20% of samples were included in statistical testing. This approach was selected to capture the compositional nature of the data while accounting for the substantial overall microbiome depletion induced by antibiotic treatment, which violates the assumptions of count-based differential abundance methods that assume constant total abundance across samples. The inulin cohort was analyzed longitudinally, with DESEQ2 to compare pre- vs post-treatment in the inulin-treated mice. Traditional filtering of reads present five times in 10% or more of samples was applied, with default DESEQ2 count normalization and dispersion estimates. Statistical significance was determined using the Wald test with Benjamini-Hochberg FDR correction, with adjusted *P* values less than 0.1 considered significant for discovery purposes. The postcounts transformation was used for log2 fold change shrinkage to improve effect size estimates for low-count ASVs. These differing approaches were used due to differences in the types of interventions and study designs, with inulin being a more subtle and targeted influence, making a more in-depth and count-based framework more appropriate. Alpha diversity metrics (Shannon index, Simpson index, and observed ASVs) were calculated after filtering to retain only ASVs present in ≥10% of samples. This prevalence filter removes rare taxa that likely represent sequencing artifacts, contaminants, or transient colonizers rather than established community members, which is particularly important when comparing communities with vastly different total bacterial loads (e.g., antibiotic-depleted vs control microbiomes).

### Functional pathway prediction and analysis

Predicted metagenomes from the data sets were generated using PICRUSt2 v2.5.0 (24, 26) for both cohorts, with default parameters. Prior to analyses, predicted pathways present in greater than 10% of samples with mean relative abundance ≥1 × 10^−^⁵ and more than 10 total read counts were retained, and centered log-ratio (CLR) transformed prior to analysis. Base R linear models were used to identify biological pathways significantly depleted by antibiotic treatment. By including sex as a covariate, we controlled for potential confounding factors and characterized the resulting loss of metabolic function. For the inulin cohort, pathway abundances were analyzed with MaAsLin2 v1.0 (27), with fixed effects and an interaction for diet and time, sex as a covariate, and a random intercept for subject ID. *P* values were FDR corrected (Benjamini-Hochberg, *q* < 0.10).

Inulin supplementation altered pathways from multiple functional categories (see Data S1 for complete results), but we focused our primary analysis/reporting on carbohydrate metabolism pathways for several reasons: (i) mechanistic relevance: SCFAs produced from microbial carbohydrate fermentation are well-established gut-brain signaling molecules (9, 15) and represent the most direct link between dietary fiber/microbiome composition and host physiology; (ii) complementary loss/gain-of-function evidence relative to the ABX cohort: ABX treatment depleted glucose degradation pathways (β = −2.28, *q* = 0.018), while inulin treatment induced the exact inverse effect (β = +2.28, *q* = 0.018), suggesting that fermentative capacity specifically drives exercise-related microbiome effects; (iii) metabolomic validation: cecal SCFA measurements from our prior work confirmed that inulin treatment elevated cecal butyrate (21, 22); (iv) effect magnitude: carbohydrate pathways showed the largest bidirectional effects and highest proportion of significant changes in both cohorts. This focused approach maintains the scope of the publication and reduces multiple testing burden.

### Neurotransmitter assay

After the VWR period, mice were run on a motorized treadmill for 1 h prior to sacrifice. In this hour, they were started at 25 m/min, and the speed was increased by 1 m/min for the first 10 min up to 35 m/min, where they were kept for the remaining 50 min at a 5% grade. This bout of exercise served as a controlled stimulus to account for the sensitive and dynamic nature of dopamine (DA) release. The protocol was adapted from a similar protocol that has been successfully used to increase striatal DA concentrations for up to 15 min after cessation of exercise (7). After treadmill running, mice were euthanized via CO_2_ asphyxiation, and brain tissue was rapidly removed and snap frozen. Samples were stored at −80°C. Frozen tissues were weighed and bead homogenized for 3 min at maximum speed in 1 mL of methanol with 10 μL of deuterated DA internal standard (Cambridge Isotope Laboratories, Inc.). Samples were then refrigerated at 4°C for 5 min, then centrifuged at 15,000 RPM at 4°C for 10 min. A total of 500 μL of supernatant was then extracted and dried down with a speed vac.

Brain sample extracts were analyzed by 5500 QTRAP (SCIEX, Redwood City, CA) liquid chromatography-tandem mass spectrometry. Subsequently, samples were injected (20 μL) into the Agilent 1200 system and equipped with a Phenomenex Kinetex C18 100A column (4.6 × 100 mm, 2.6 μm) with mobile phase A (0.1% formic acid in water) and mobile phase B (0.1% formic acid in acetonitrile). The flow rate was 0.4 mL/min. Mass spectra were acquired under both positive and negative electrospray ionization (ESI) with the ion spray voltage at −4,500/5,500 V. The source temperature was 500°C. The curtain gas, ion source gas 1, and ion source gas 2 were 35, 65, and 55 pounds per square inch, respectively. Target compounds were detected by multiple reaction monitoring (Table S2). Calibration curves were generated for the range of 0.02–1,000 ng/mL. Software Analyst 1.7.1 (Agilent) was used for data acquisition and analysis. Resultant concentrations were corrected to sample wet weight.

While most metabolites selected for the panel were chosen based on their biochemically “adjacent” relationship to DA, a few other excitatory and inhibitory neurotransmitters related to locomotor activity, arousal, and perception of fatigue were also included.

### Serum corticosterone

Immediately following the treadmill exercise stimulus, blood was collected via cardiac puncture, and serum was isolated. Corticosterone concentrations were analyzed in serum via an ELISA kit (Enzo Life Sciences, Farmingdale, NY) following the manufacturer’s instructions.

### General statistical approach

**Fig 1:**
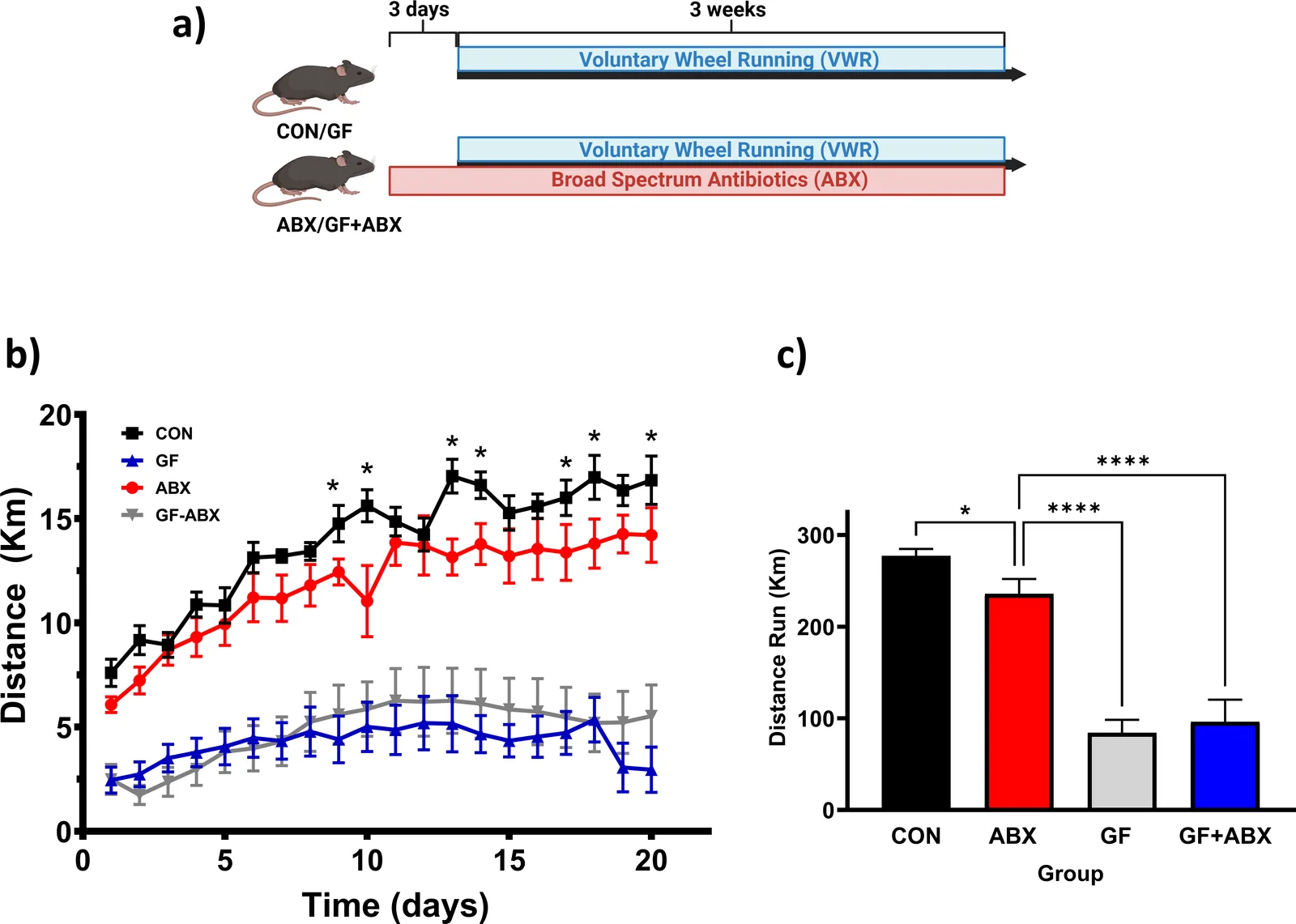
Broad-spectrum antibiotic treatment reduces wheel running acquisition in control specific pathogen free (CON), but not GF, mice. (**a**) Experimental design schematic. (**b**) Daily running volume for mice during the 20-day period. (c) Cumulative running distance over the 20-day acquisition period. In panel **b**, * indicates significantly lower than CON (*P* < 0.05) and **** indicates significantly lower than CON and ABX via Tukey’s HSD (*P* < 0.0001). In panel **c**, * indicates significantly lower than CON via Welch’s *t*-test. *N* = 9, 9, 10, and 9 for CON, ABX, GF, and GF-ABX, respectively.

Wheel running data were not normally distributed in some instances. A square transformation was used for negatively skewed data (Fig. 1b) and a square root transformation for positively skewed data (see Fig. 4b). Data are all presented in figures untransformed, but regression models were run on transformed versions. Considering occasional equipment failure (i.e., battery charge loss), which resulted in missing data, a repeated-measures linear mixed-effects regression was used following transformation to detect main effects and interactions. Treatment groups and time were designated as fixed effects with mouse ID as a random effect. Where main effects were detected, pairwise comparisons were conducted with a Student’s *t*-test between all treatment groups for each day, and in the case of multiple comparisons, *P* values were adjusted via Benjamini-Hochberg FDR. In cases where equipment failed multiple times, data from that mouse were excluded from the analysis due to uncertainty of accuracy of data and stunted acquisition. Data without repeated measures, such as striatal metabolites and serum corticosterone, were compared by either one- or two-way ANOVA with subsequent Fisher’s least significant difference (Fisher’s LSD) or Tukey honestly significant difference (Tukey HSD). All stats were conducted in RStudio version 2023.03.1 with R version 4.1.2. To assess associations between striatal neurochemical concentrations and voluntary wheel running behavior, Spearman correlations were computed between dorsal striatal dopamine and histamine concentrations and cumulative VWR distance within each treatment group separately. Cumulative VWR distance (sum of all available daily distance values per animal) was used as the behavioral summary measure, as neurochemical tissue samples were collected immediately following a controlled exercise bout at the end of the full VWR period. Benjamini-Hochberg FDR correction was applied across all group-by-neurochemical comparisons (14 tests total). Animals were included only if paired VWR and neurochemical data were both available (*n* = 66 across seven groups). Potential within-group outliers were flagged using the 1.5 × interquartile range (IQR) rule and are annotated in Fig. S7; all animals were retained in analyses regardless of outlier status. Sample sizes for striatal neurochemistry, serum corticosterone, and microbiome analyses varied modestly across measures due to tissue availability at collection and resource constraints (e.g., per-sample assay and sequencing costs), which limited the number of samples processed for some secondary outcomes; specific *n* values are reported in figure legends and Table 1.

**TABLE 1:** Excitatory and inhibitory neurotransmitter responses in the striatum following a controlled bout of exercise^*a*^

Treatment	L-DOPA (ng/g)	Dopamine (ng/g)	DHPAA (ng/g)	HVA (ng/g)	Norepinephrine (ng/g)	Epinephrine (ng/g)	Histamine (ng/g)	GABA (µg/g)	Serotonin (ng/g)
GF	**147.2 ± 11.1**	**1,101.8 ± 143.2**	260.0 ± 37.4	503.3 ± 85.2	**27.1 ± 2.8**	4.9 ± 1.1	372.8 ± 53.4	27.2 ± 3.9	70.5 ± 7.3
ABX	111.3 ± 9.3	713.1 ± 89.3	264.4 ± 22.2	573.6 ± 45.7	18.2 ± 1.77	4.7 ± 0.9	467.4 ± 35.9	29.6 ± 6.8	64.0 ± 4.1
ABX + SCFA	140.0 ± 18.8	740.7 ± 116.6	244.3 ± 27.7	624.7 ± 73.0	23.9 ± 3.5	4.2 ± 0.7	422.5 ± 33.2	29.9 ± 1.8	65.0 ± 2.7
SCFA	124.5 ± 24.3	841.7 ± 85.2	306.1 ± 39.1	596.8 ± 53.8	17.4 ± 2.2	6.2 ± 0.72	545.4 ± 78.6	**35.8 ± 1.5**	66.5 ± 5.5
CON	98.6 ± 11.7	624.7 ± 102.4	203.9 ± 26.0	511.6 ± 71.0	20.6 ± 2.3	3.8 ± 0.7	434.8 ± 43.7	27.0 ± 1.5	62.6 ± 1.8
INU	90.0 ± 7.6	669.5 ± 48.8	**288.3 ± 19.3**	571.3 ± 43.5	23.8 ± 2.9	6.3 ± 1.0	**692.5 ± 55.0**	30.6 ± 2.5	69.3 ± 6.9

^*a*^ Shaded columns are for metabolites which are considered “excitatory,” and unshaded columns are for those considered “inhibitory.” Boldfacing indicates significant difference from CON (*P* < 0.05). *n* = 8–10/group.

## RESULTS

### An intact microbiota contributes to optimal voluntary wheel running acquisition

Antibiotic-induced depletion of the gut microbiota or germ-free status reduces VWR acquisition (*P* = 0.04 for CON/ABX, *P* < 0.0001 for GF) mice (Fig. 1b), consistent with our previous observations (8). ABX significantly reduced total daily running on 7 days, while GF status resulted in significantly reduced running for every day in the trial period (Fig. 1b). Next, we used an identical experimental design in GF mice to confirm the observed effects are indeed due to microbial depletion and not independent non-anti-microbial effects of ABX, which have been displayed in skeletal muscle of specific pathogen free (SPF) and GF mice (19). We found no significant difference in wheel running between GF and GF + ABX mice (*P* = 0.77; Fig. 1b), indicating that observed effects in SPF mice are indeed due to depletion of the gut microbiota and not some side effect of ABX (Fig. 1c). Given that both GF and GF + ABX mice still accumulated roughly 100 km running volume over the trial period, the absence of a further reduction in GF + ABX relative to GF is likely not attributable to a floor effect. In a separate pilot study, we performed a longer version of the experiment where we began ABX at day 27 of VWR, which is well into the maintenance phase (12, 28). We found no reduction in daily VWR in ABX-treated mice (*P* = 0.78; Fig. S1a and b). These findings establish microbial causality, showing that an intact gut microbiota is needed for optimal VWR acquisition and not maintenance.

### Antibiotic perturbation of the gut microbiota impairs predicted fermentation capacity

**Fig 2:**
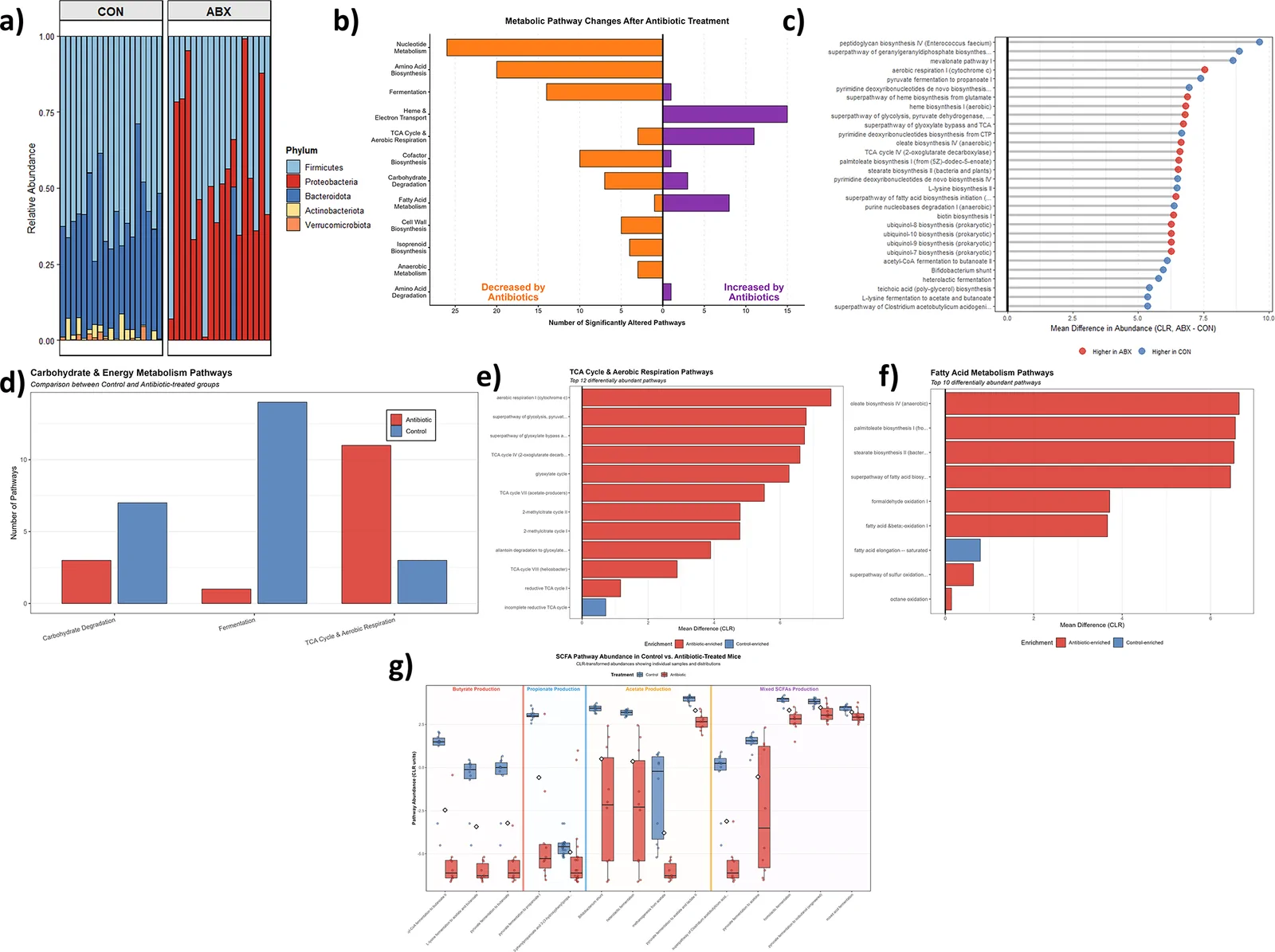
Antibiotic perturbation of the gut microbiota enriches predicted pathways for aerobic respiration and depletes pathways related to fermentation/SCFA production. (**a**) Relative abundance of major phyla in mice in both treatment groups. (**b**) Number of predicted metabolic pathways significantly increased in respective treatment groups relative to one another, grouped in broader functional categories. (**c**) Top differentially abundant pathways between groups and respective mean differences, colored by treatment group displaying elevated content. (**d**) Number of elevated pathways in each group related to carbohydrate processing. (**e and f**) Individual metabolic pathway differences (shown by mean difference) associated with fatty acid metabolism and aerobic respiration in ABX mice. (**g**) Absolute abundance of pathways associated with SCFA metabolism identified as significantly different between groups.

To identify microbial metabolic functions associated with reduced wheel running, we used PICRUSt2 for functional metagenome prediction to outline metabolic deficiencies imparted by antibiotic perturbation in our previous data set displaying ABX-induced reduction in VWR (8). The same antibiotic cocktail was used, and experiments were conducted in the same animal facility. β-Diversity analysis via weighted UniFrac indicated that baseline microbiomes between groups were equivalent (PERMANOVA *P* = 0.395), but antibiotic treatment potently disrupted the existing community (PERMANOVA *P* = 0.001, *R*^2^ = 0.456; Fig. S3). Reduced fecal DNA yield in this same cohort following antibiotic treatment has been previously reported (8), consistent with the compositional and diversity-based depletion evidence presented here. Compositionally, antibiotic treatment dramatically altered the microbiome at the phylum level and reduced alpha diversity across three metrics (Fig. 2a and S4). Proteobacteria (facultative anaerobes) expanded from 2.3% ± 1.2% to 47.8% ± 8.3% (20.8-fold increase, FDR-adjusted *P* < 0.001), while Bacteroidota (obligate anaerobes) declined from 52.1% ± 6.4% to 9.8% ± 4.2% (5.3-fold decrease, FDR-adjusted *P* < 0.001). Accordingly, the predicted metagenome was altered drastically, with 226 metacyc pathways significantly altered by antibiotic treatment (Fig. 2b). Specifically, ABX enriched pathways contributing to aerobic respiration, while depleting pathways related to carbohydrate degradation and fermentation (Fig. 2c through f). This metabolic shift reflects expansion of facultative anaerobes and depletion of obligate anaerobic fermenters, consistent with reduction in pathways supporting SCFA production (Fig. 2g). These findings suggest that fermentation capacity may be an important determinant in the microbiota’s influence on exercise motivation.

### SCFA supplementation restores VWR acquisition in microbiota-depleted mice

**Fig 3:**
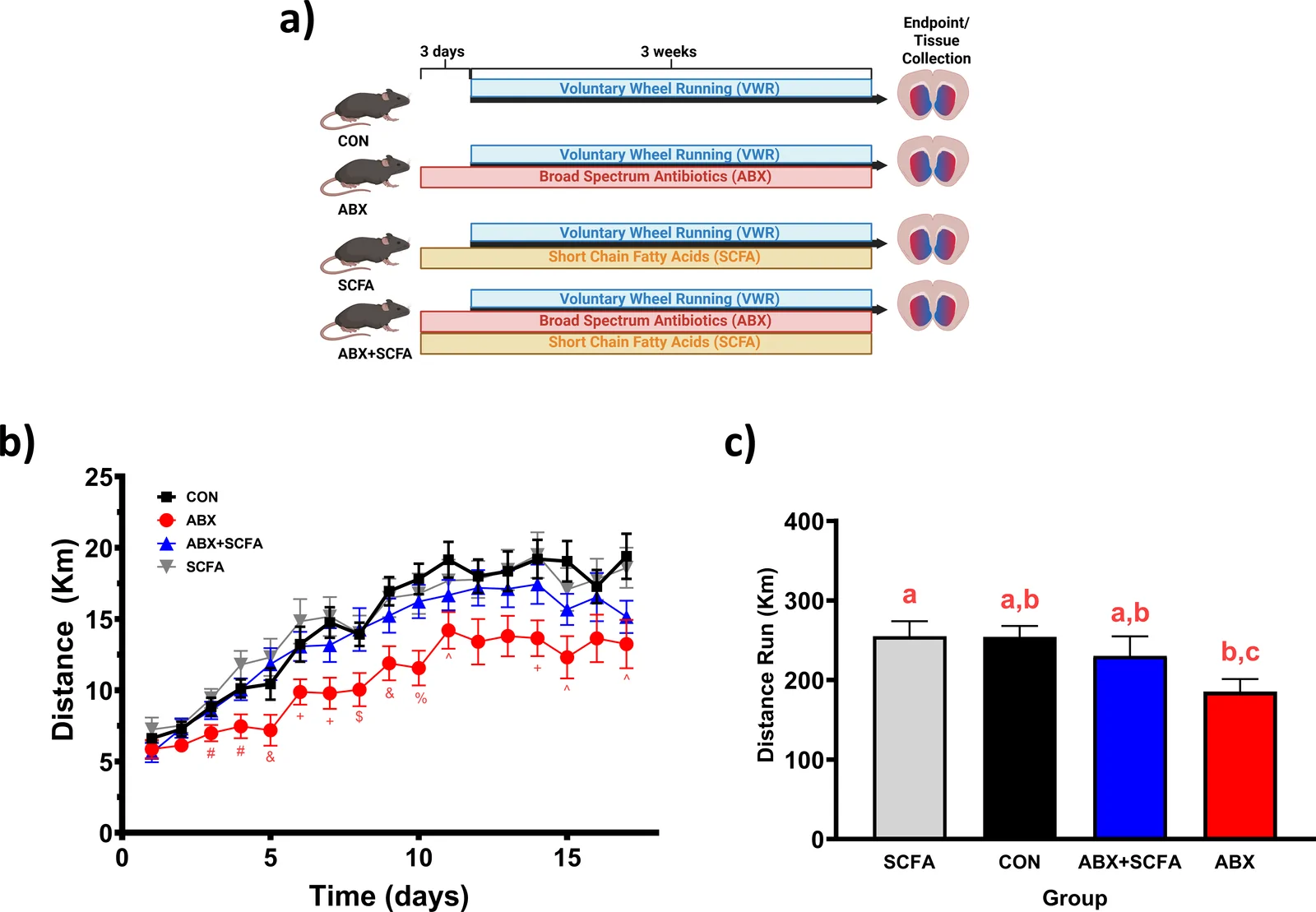
Concurrent supplementation of SCFA restores VWR acquisition during microbiota depletion. (**a**) Study design schematic for experiments. (**b**) Daily running volumes of mice throughout the 17-day trial period. (**c**) Total running volumes throughout the 17-day period. Symbols in panel b indicate significant differences via Dunnett’s *D* (*P* < 0.05) between the following: #=, ABX and SCFA; &, ABX and SCFA/ABX + SCFA; +, ABX and SCFA/CON; $=, ABX and ABX + SCFA; %=, ABX and SCFA/CON/ABX + SCFA; ^, ABX and CON. * in panel b indicates that INU mice ran significantly more than CON mice on that respective day (*P* < 0.05).

To examine whether supplementing SCFA exerts additive or restorative effects on VWR activity in CON or ABX mice, we administered a mixture of acetate, propionate, and butyrate in drinking water to both treatment groups in a 2 × 2 design (Fig. 3a). We found that supplemental SCFA returned VWR activity in ABX mice to levels equivalent to CON mice (ABX *P* = 0.0001, ABX × SCFA *P* = 0.03, Fig. 3b and c), but they were not additive in that they did not increase running in CON mice with intact microbiotas (SCFA *P* = 0.77; Fig. 3b and c). This finding suggests that SCFA supports healthy acquisition of VWR as a habit and may play a role in the motivation to engage in exercise.

### Dietary inclusion of prebiotic inulin enhances predicted microbiota fermentation capacity and VWR acquisition

**Fig 4:**
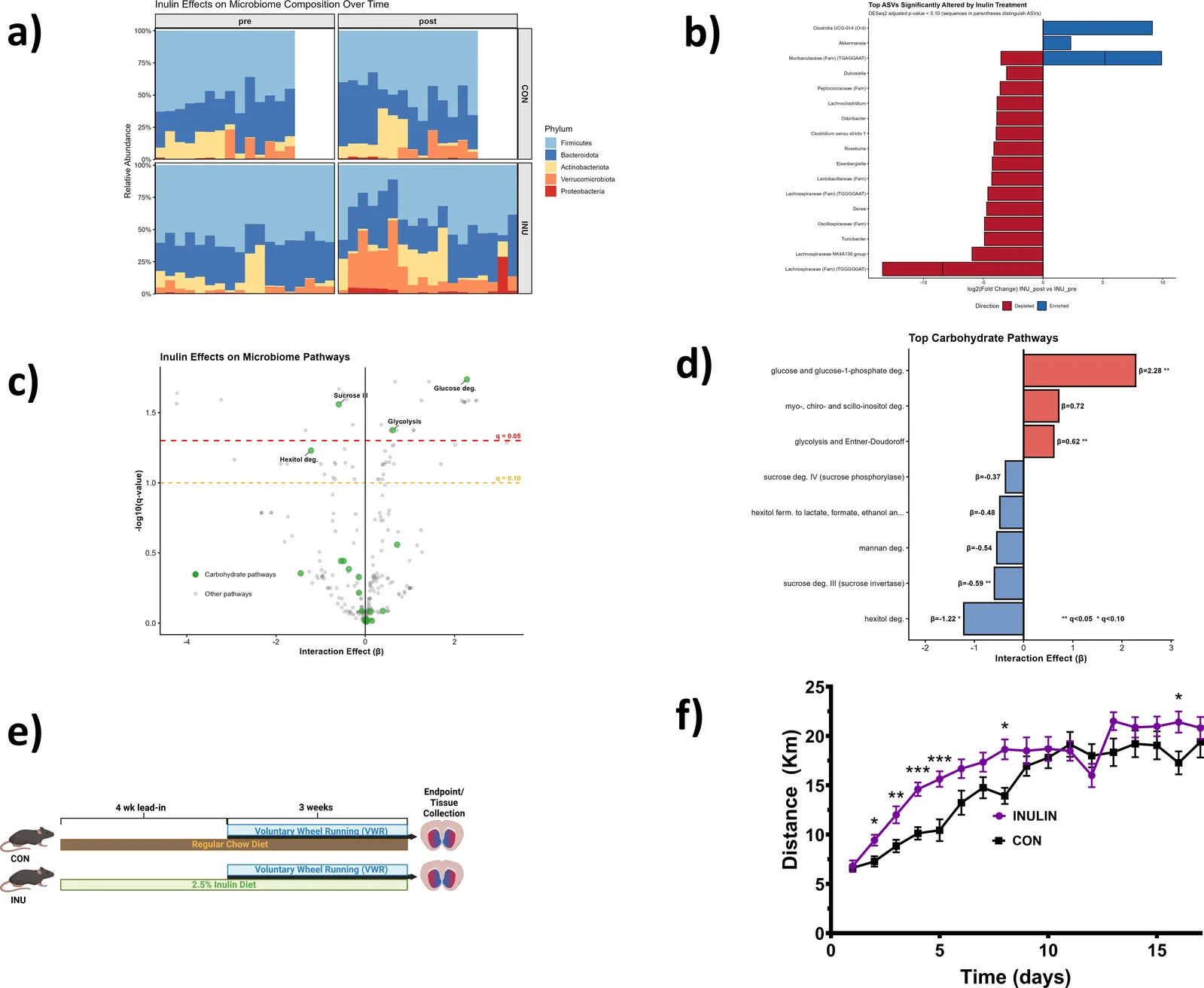
Dietary inclusion of prebiotic inulin enhances predicted microbiota fermentation capacity and VWR acquisition. (**a**) Relative abundance of major phyla in control and inulin-treated mice before and after 8-week intervention. (**b**) Top ASVs significantly altered by inulin treatment after inulin intervention. (**c**) Volcano plot of pathways altered by the dietary intervention period (time × diet interaction). (**d**) Interaction coefficients (effect size for diet × time interaction effect) for carbohydrate-related pathways altered by inulin treatment. (**e**) Study design for wheel running experiment. (**f**) Daily running distances for control mice and mice fed the inulin-enhanced diet. *P < 0.05 or *q* < 0.10, **P < 0.01 or *q* < 0.05, and ***P < 0.001.

To examine a more readily translatable approach, we utilized PICRUSt2 for metagenomic inference on a 16S sequencing data set from our prior publication, where we treated mice with the same diet used here for 8 weeks, enhanced with 2.5% of the prebiotic and butyrogenic fiber, inulin (23, 29). β-Diversity analysis via weighted UniFrac revealed a modest but significant difference in baseline community structure between mice subsequently assigned to control or inulin diets (*R*^2^ = 0.126, *P* = 0.038), which increased over the 8-week intervention (post-treatment: *R*^2^ = 0.200, *P* = 0.012), with a significant diet × timepoint interaction (*R*^2^ =0.069, *P* = 0.007) confirming that dietary divergence accumulated over time beyond the modest baseline difference (Fig. S5). Taxonomic analyses revealed substantial restructuring, with enrichment of specialized fiber-degrading taxa (*Akkermansia muciniphila*, *Muribaculaceae* family members) and depletion of several *Lachnospiraceae* species including the butyrate producer *Roseburia* (*P*_adj_ < 0.05, Fig. 4a and b). Despite this apparent loss of canonical butyrate producers, we have previously shown this diet to elevate cecal butyrate concentrations (23, 29), indicating functional compensation by the restructured community. Additionally, inulin reduced alpha diversity (Shannon, Simpson, and observed ASVs; all *P* < 0.014; Fig. S6), suggesting that microbiota function can be resilient to taxonomic reorganization.

Functionally, inulin effects were most pronounced in predicted pathways related to carbohydrate degradation (Fig. 4c). Inulin induced significant upregulation of glucose/glucose-1-phosphate degradation (β = 2.28, *q* = 0.018), and glycolysis/Entner-Doudoroff pathways (β = 0.62, *q* = 0.042; Fig. 4d). These changes demonstrate active carbohydrate utilization, representing a functionally inverse aspect in relation to changes observed due to antibiotic treatment. Building on this, we pretreated mice with four weeks of a 2.5% inulin-enhanced diet prior to VWR acquisition (Fig. 4e) and found that it significantly increased wheel running above that of CON on 11 days throughout the acquisition period (*P* = 0.04 for main effect, *P* < 0.0001 for inulin × time interaction, FDR *P* < 0.05; Fig. 4f). Collectively, these findings suggest that carbohydrate utilization by the gut microbiota not only supports baseline VWR habit acquisition but also that interventions that modulate this function can potentiate motivation and VWR engagement.

### Microbiome modulation produces context-dependent dysregulation of exercise-induced corticosterone signaling

**Fig 5:**
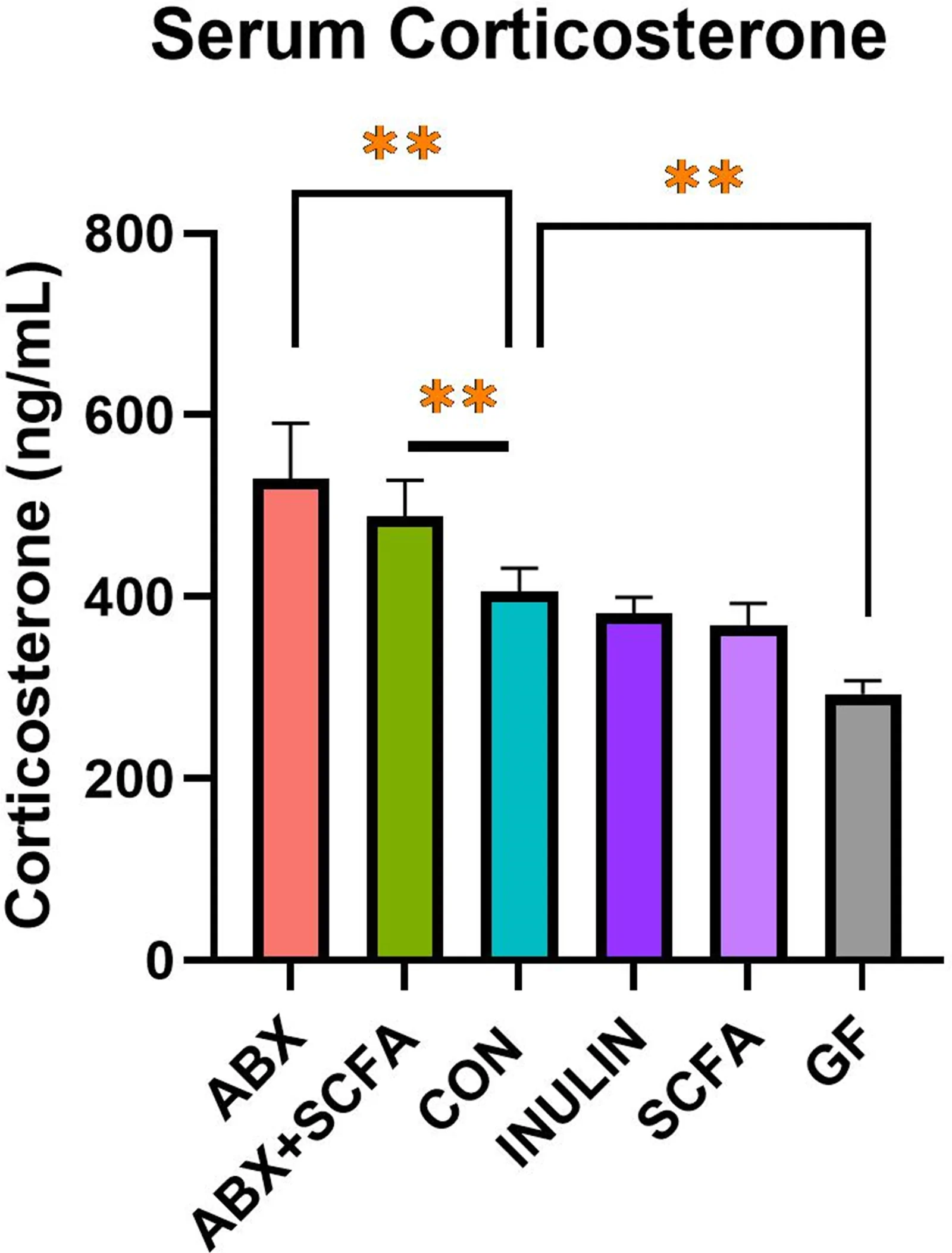
Microbiome modulation produces context-dependent dysregulation of exercise-induced corticosterone release. Serum corticosterone concentrations in mice of each treatment group following the controlled bout of forced exercise. *n* = 7/group for all groups aside from SCFA, for which *n* = 6. Data were analyzed by one-way ANOVA followed by Fisher’s LSD. ** indicates *P* < 0.01.

**Fig 6:**
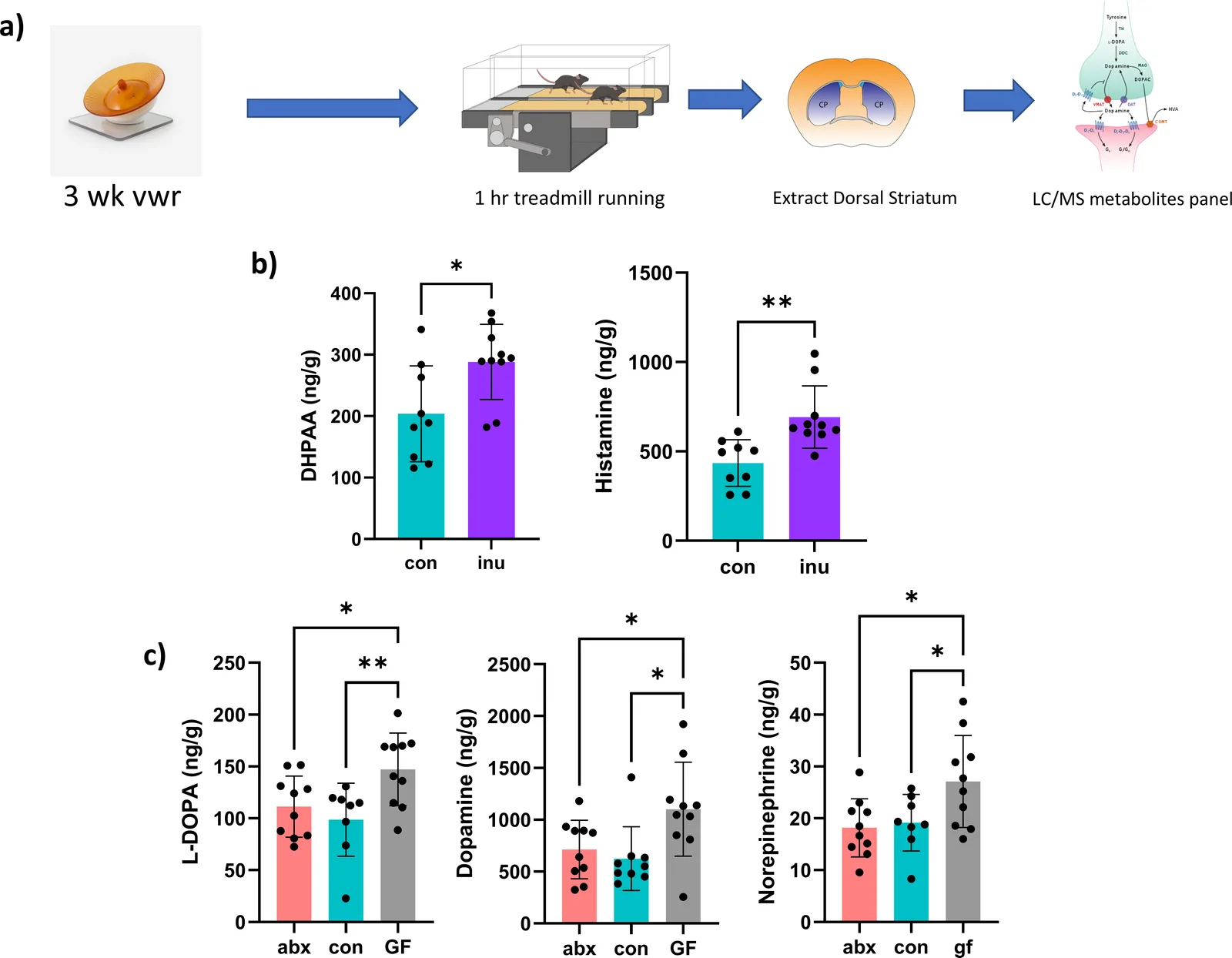
Excitatory and inhibitory neurotransmitter responses in the striatum following a controlled bout of exercise. (**a**) Study design used to extract and quantify metabolites in the dorsal striatum. (**b**) Metabolites significantly increased in the striatum of inulin-fed mice. (**c**) Metabolites significantly increased in the striatum of GF mice following the controlled bout of exercise. In panels **b** and **c**, dots represent individual values with the transparent bars showing group means. * indicates significantly different from all other groups (*P* < 0.05); ** indicates *P* < 0.01). *N* = 10/group for all groups.

To examine whether microbial effects on VWR acquisition involve neuroendocrine signaling, we measured concentrations of corticosterone, the primary murine stress hormone, following a controlled bout of exercise. Manipulation of the microbiome produced bidirectional dysregulation of corticosterone responses to exercise (Fig. 5). Acute depletion via ABX increased post-exercise corticosterone (both ABX and ABX + SCFA, *P* < 0.01), while germ-free development decreased it (*P* < 0.01). This divergence of effects between acute depletion and lifelong absence reveals complex temporal dynamics in microbiome-HPA axis interactions. Conversely, despite reduced corticosterone, germ-free mice exhibited elevated striatal catecholamines (dopamine, L-DOPA, and norepinephrine; Fig. 6c), indicating dysregulated coupling of sympathetic-adreno-medullary (SAM) and HPA axis activity (13, 30–32). This exercise-specific dissociation contrasts with typical stress phenotypes in germ-free mice, which typically display exaggerated HPA axis responses to non-exercise stressors, suggesting unique requirements for microbiome-dependent neuroendocrine integration of metabolic signals. These findings suggest that the gut microbiota is required for optimal exercise engagement and coordinated neuroendocrine response to exercise stress, with important contributions to the development of proper SAM-HPA axis integration.

### Striatal neurochemistry following a controlled bout of exercise

To explore potential neurochemical mechanisms underlying microbiome-dependent VWR acquisition effects, we quantified neurotransmitters in the striatum following a controlled bout of treadmill exercise (Fig. 6a). Inulin pretreatment, which enhanced microbial fermentative capacity and VWR activity, increased striatal concentrations of the DA catabolite DHPAA, as well as histamine (*P* = 0.02 and 0.002, respectively; Fig. 6b). Histamine modulates excitatory inputs in striatal medium spiny neurons (33) and potentiates the effects of dopamine receptor agonists on locomotor behavior, suggesting a potential neurochemical mechanism underlying inulin’s behavioral effects (34). GF mice displayed significantly increased concentrations of DA and its immediate precursor, L-DOPA (*P* < 0.05; Fig. 6c). However, they also displayed a significant increase in norepinephrine, which is a key component of stress responses and is believed to contribute to the perception of fatigue (35, 36) (Fig. 6c). While ABX and SCFA treatment did not affect any excitatory molecule concentrations, SCFA treatment without ABX did result in higher concentrations of γ-aminobutyric acid, the chief inhibitory neurotransmitter in the CNS (*P* = 0.01; Table 1). These results suggest that microbiome modulation influences multiple processes of neurotransmission, though receptor-specific manipulations are required to draw causal conclusions. The full list of data for all metabolites and treatment groups can be found in Table 1.

To assess whether striatal neurochemical concentrations were associated with running behavior, we computed within-group Spearman correlations between dorsal striatal dopamine and histamine and cumulative VWR distance (Table S3; Fig. S7). Striatal dopamine was significantly correlated with cumulative running distance in ABX-treated mice (ρ = 0.867, *P* = 0.001, FDR-adjusted *P* = 0.016) but not in any other group, including germ-free mice (ρ = −0.103, *P* = 0.777). Striatal histamine showed a significant negative correlation with cumulative running distance in ABX + SCFA-treated mice (ρ = −0.794, *P* = 0.006, FDR-adjusted *P* = 0.043), with no significant associations in remaining groups; a non-significant positive trend was observed in inulin-treated mice (ρ = 0.491, *P* = 0.150).

## DISCUSSION

In the present study, we demonstrate that the predicted fermentation capacity of the gut microbiota influences both exercise habit acquisition and the integration of HPA axis signaling during metabolic stress. Using complementary approaches modeling microbiome absence, depletion, rescue, and enhancement, these data support the hypothesis that microbiome metabolic function influences behavioral outcomes. Additionally, we reveal context-dependent dysregulation of exercise-induced HPA axis responses due to depleted microbiota: acute antibiotic depletion increased post-exercise corticosterone, while germ-free status decreased it, despite elevated striatal catecholamines. These findings in mice establish gut fermentation capacity as a modifiable determinant of motivated behavior and identify microbiome-dependent coupling of SAM and HPA axes as a novel mechanism linking gut metabolism to exercise motivation.

Functional metagenomic prediction suggests that compositional microbiome modulation produces concurrent metabolic shifts in fermentation pathways. Consistent with the depletion of obligate anaerobes and expansion of facultative anaerobes, antibiotic-induced microbiome depletion shifted the predicted metagenome away from pathways for carbohydrate fermentation while enriching capacity for aerobic respiration. Conversely, prebiotic inulin consumption specifically enhanced anaerobic pathways for glucose utilization, indicating increased fermentative substrate utilization. The correlation between predicted fermentation capacity and exercise behavior, along with experiments showing SCFA supplementation can rescue this behavior, suggests that microbial metabolic output plays an important role in these effects. While PICRUSt2 provides predicted function rather than measured capacity, the functional predictions align with prior observations and established effects of antibiotics and prebiotics on fecal SCFA (23, 29). This function-centric approach reveals microbiome fermentation capacity to be a functionally assessable and modifiable trait that associates with motivated behavior, identifying a potential therapeutic target.

Beyond metabolic mechanisms, our findings reveal a novel dimension of physiological microbiome influence: neuroendocrine integration during exercise stress. The finding of a depleted corticosterone response following forced treadmill exercise is seemingly contradictory to a body of literature that displays that germ-free mice exhibit exaggerated stress responses and corticosterone release following non-exercise stimuli (36–38). However, when interpreted along with the finding of exaggerated responses to exercise in striatal neurochemicals (dopamine, L-DOPA, and norepinephrine), this suggests that this effect may be specific to exercise-induced HPA axis activation and/or corticosterone release. The uncoupling of activation between the SAM and HPA axes in GF mice specific to conditions of high metabolic demand suggests that developmental defects result in ineffective integration of metabolic signals in HPA axis regulation. Along these lines, GF mice exhibit defects in multiple processes that modulate HPA axis activation. In addition to abnormal regulation of blood glucose concentration and skeletal muscle (8, 39, 40), dysregulations in the release of metabolic hormones such as ghrelin and leptin have also been documented (41). With that said, impaired steroidogenesis in the small intestine may also play a role in this phenomenon, a process which is subject to influence by the gut microbiota (42).

Given that post-exercise corticosterone was increased in ABX mice, our findings demonstrate that the microbiota and SCFA are not only crucial for the maturation of the HPA and SAM axes but also play acute regulatory roles in HPA axis activity in the context of exercise. While circulating corticosterone concentrations do not directly correlate with striatal dopamine levels under all conditions, they have been shown to influence wheel-running behavior (34). In conjunction with recent evidence that glucocorticoid signaling affects muscular remodeling and adaptation, these findings open new avenues for exploring the interplay between the gut-brain and gut-muscle axes through the lens of stress biology for the improvement of general health and physical fitness (43, 44). To clarify the mechanistic basis of this exercise-specific uncoupling, future studies characterizing upstream (e.g., hypothalamic corticotropin-releasing hormone (CRH) expression) and downstream (e.g., adrenal sensitivity to andrenocorticotropic hormone (ACTH)) readouts of HPA axis activity represent an important direction.

SCFA have been identified as transducers in gut-brain signaling due to their role as bioenergetic substrates and regulators of gene expression, primarily through FFAR signaling and histone deacetylase inhibition (45). Of the many means of gut-brain communication, available evidence suggests that sensory afferent signals through gut-innervating spinal neurons appear to be the most influential on striatal circuits mediating exercise engagement and motivation (7, 46). Colonic L cells, which express FFAR2 and FFAR3, may be coupled with sensory nerves in the gastrointestinal (GI) tract to initiate bioenergetic status-dependent afferent signaling to central motivation pathways via FFAR2-induced release of the incretin glucagon-like peptide 1 (47, 48). The present data confirm that SCFA can be supplemented to act on gut-brain circuits which influence motivation, but future studies supplementing individual SCFA are needed to determine which SCFA can influence this phenotype. Additionally, while administration of the SCFA cocktail to mice with intact microbiota did not increase wheel running above baseline, dietary inulin supplementation was capable of enhancing VWR activity. This discrepancy may be explained by either (i) the duration of pretreatment or (ii) differences between prebiotic (e.g., inulin) and postbiotic (e.g., SCFA) interventions.

Inulin consumption is known to elevate SCFA levels (23, 29), and higher dietary fiber intake has been associated with improved physical fitness markers in individuals over 55 years old (49). These findings suggest that long-term dietary patterns and fiber intake may be more practical approaches in enhancing exercise motivation. Additionally, the concurrent increase in post-exercise striatal histamine in inulin-treated mice points to a potential histamine-dependent mechanism responsible for their elevated wheel-running activity. Prior evidence that histamine agonists can amplify the locomotor effects of dopamine agonism supports this hypothesis (34). Finally, supplementation with heat-killed *Lactobacillus brevis* SBC8803 also led to increased VWR over a 26-day period (50), indicating that microbial metabolites alone do not fully explain microbial influence on wheel-running acquisition. Taken together, current evidence suggests that consideration of both the type and duration of microbiota-targeted interventions is important when evaluating their impact on exercise motivation.

Prior work established that acute microbiome depletion reduces striatal dopamine during exercise, and dopamine concentrations are critical in driving movement and behavior (7, 51). However, the contributions of other neurotransmitter systems in microbiome-dependent effects in this context have remained unexplored. We examined a panel of striatal neurochemicals to identify potential neural mechanisms underlying microbiome-dependent exercise behavior and examined their correlations with wheel running totals to explore relevance to motivation. Notably, the inulin-induced increase in VWR acquisition co-occurred with increased striatal histamine after exercise, along with a non-significant positive correlative trend with running distance (ρ = 0.491, *P* = 0.150). These findings suggest histaminergic neuromodulation as a potential mechanism warranting further exploration beyond this limited sample. Histamine modulates medium spiny neuron excitability and potentiates the locomotor effects of dopaminergic stimulation (33, 34), suggesting a plausible pathway for dietary inulin to influence exercise motivation. It should also be noted that a significant negative correlation was observed between histamine and running distance in ABX + SCFA-treated mice, which should be interpreted with caution due to the limited sample size and presence of an outlier. Whether this reflects a genuine inverse relationship specific to the treatment condition or experimental artifact requires further study.

Germ-free mice exhibited elevated catecholamines after exercise, despite reduced exercise activity, which may reflect physiological factors such as greater perceived effort or other developmental defects, but may also be due to methodological artifact. The LC-MS methods employed here quantified total tissue dopamine, meaning that the relative contributions of intracellular vesicular stores, cytoplasmic pools, and synaptic dopamine, which drives neuronal excitation, cannot be differentiated. As a result, elevated tissue dopamine may be caused by either increased synthesis, increased afferent dopaminergic input from nigrostriatal projections, reduced release, or reduced enzymatic degradation. Taking this into account, this result and the lack of correlation between striatal tissue dopamine and wheel-running volume in GF mice should be interpreted with caution. To resolve this ambiguity, techniques capable of measuring extracellular dopamine release dynamics directly, such as *in vivo* microdialysis or fast-scan cyclic voltammetry during voluntary wheel running, will need to be employed.

While speculative due to the aforementioned caveats, the positive correlation between striatal tissue dopamine and running distance in ABX-treated mice may suggest that antibiotic-induced disruption of gut-brain signaling may alter the normal relationship between dopamine synthesis, storage, and behavioral modulation. Importantly, neither ABX nor SCFA treatment altered striatal dopamine following exercise, suggesting that neurochemical effects observed during early habit acquisition in prior work may normalize with neuronal adaptation throughout the process of habit formation.

Although VWR does not fully capture the complexity of human lifestyles and exercise habits, it is a motivated behavior that is highly phylogenetically conserved (43, 52). Motivation is a key barrier to physical activity in sedentary individuals (5), and our findings suggest that regular consumption of dietary soluble fiber may enhance motivation during the early stages of habit formation. Since reduced activity during acquisition can persist for weeks (50), early engagement may be critical for long-term adherence. Given that existing habits improve adherence to new exercise routines (53), dietary fiber could serve as a strategy for promoting sustained physical activity in early life or sedentary individuals.

Several limitations with this work need to be considered. First, PICRUSt2 provides predicted rather than measured metabolic capacity, meaning shotgun metagenomic sequencing and targeted metabolomics would be needed to functionally validate fermentative output and identify key metabolites beyond SCFA. This gap is particularly salient in the antibiotic-treated mice; direct SCFA quantification would strengthen confidence in the proposed mechanism. Second, our neurochemistry measurements are associative; pharmacological receptor manipulation, vagotomy, and manipulations in neural circuitry are needed to establish causal mechanisms. Third, our pilot data on phase specificity (acquisition vs maintenance) require confirmation in studies with adequate sample sizes. Fourth, we focused solely on male mice, ignoring sex, a highly important biological variable in this context. Fifth, while VWR models exercise and motivated behavior, observed findings require clinical validation, including consideration of exercise modality, intensity, and human behavioral characteristics. Finally, the lack of comprehensive comparison of temporal effects during acquisition vs sustained effects after adaptation requires longitudinal studies of greater length to confirm long-term benefits.

In conclusion, our study demonstrates that gut microbiota disruption impairs early-stage exercise motivation, which can be mitigated by SCFA supplementation. However, only long-term prebiotic intake enhanced activity beyond baseline and influenced striatal neurochemical concentration. These results underscore the importance of prebiotic interventions and long-term dietary patterns in shaping exercise behavior and neural adaptation. Future research should explore the potential of prebiotics, probiotics, postbiotics, and synbiotics to enhance motivation and engagement in physical activity across diverse populations and should consider modulation of histaminergic neurotransmission as a modifiable target mechanism.

## Data Availability

The raw 16S rRNA gene sequencing data generated in this study have been deposited in the NCBI Sequence Read Archive (SRA) under BioProject accession number PRJNA1420204. Processed data tables (including metabolomics and behavioral results) are available via Zenodo at doi 10.5281/zenodo.18526012.

## SUPPLEMENTAL MATERIAL

Data S1 msystems.00876-26-s0001.xlsx PICRUSt2 MetaCyc pathway predictions and full analysis outputs for stats.

Supplemental figures msystems.00876-26-s0002.pdf Figures S1 to S7.

Supplemental tables msystems.00876-26-s0003.docx Tables S1 to S3.

ASM does not own the copyrights to Supplemental Material that may be linked to, or accessed through, an article. The authors have granted ASM a non-exclusive, world-wide license to publish the Supplemental Material files. Please contact the corresponding author directly for reuse.
