# Supplementary material for: Fermentation capacity of the gut microbiota influences exercise motivation and neuroendocrine integration
Source: mSystems. 2026 Aug 19;11(9):e00876-26. doi: 10.1128/msystems.00876-26 (PMC13595965; doi:10.1128/msystems.00876-26)
Supplement: Supplementary file 1 [file msystems.00876-26-s0002.pdf]

Figure S1

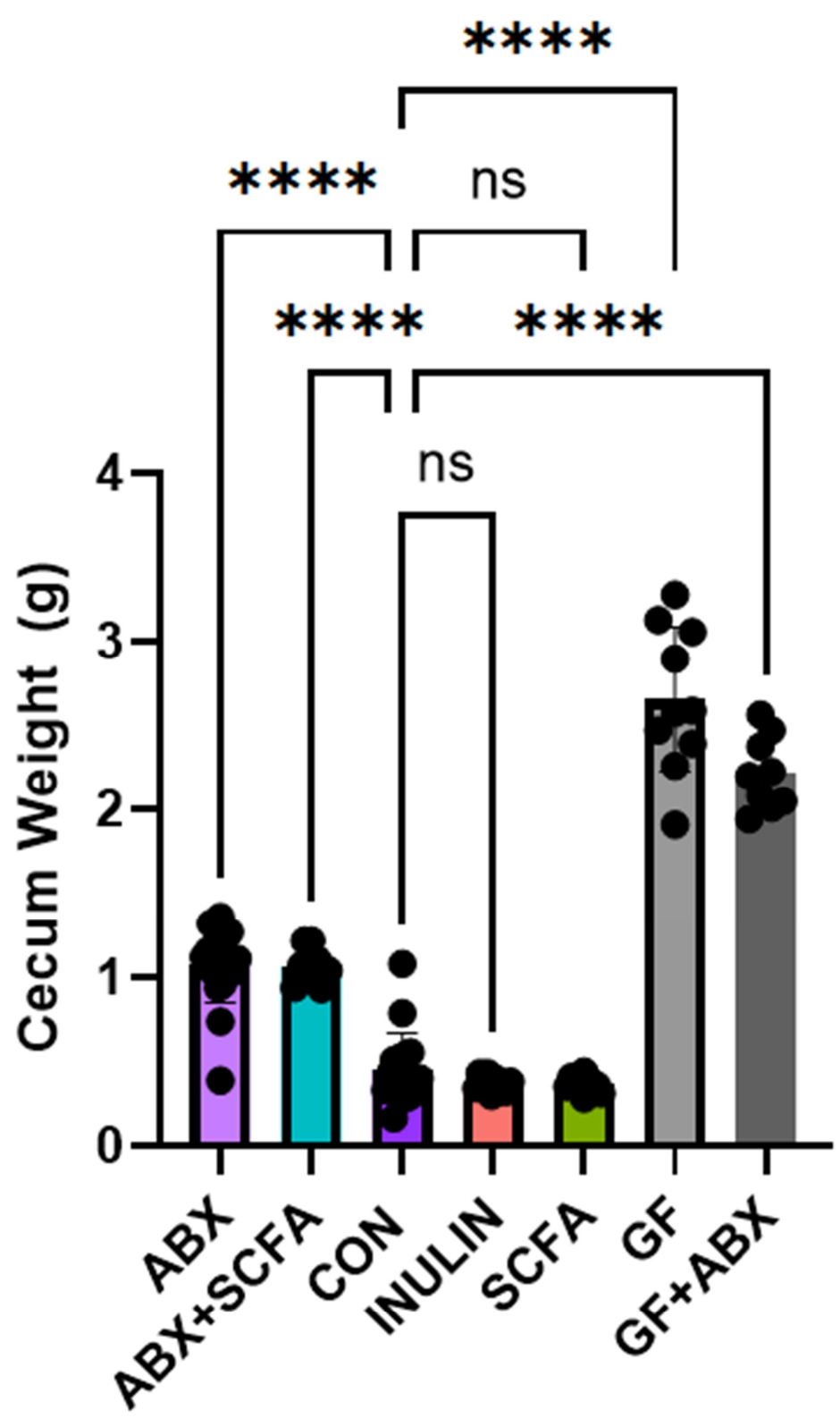

Supplemental Figure 1. Cecum weights of mice at sacrifice indicate lack of a health native microbiota following antibiotic treatment or in mice raised under germ free conditions. n=9-19/group. **** indicates p< 0.0001.

Figure S2

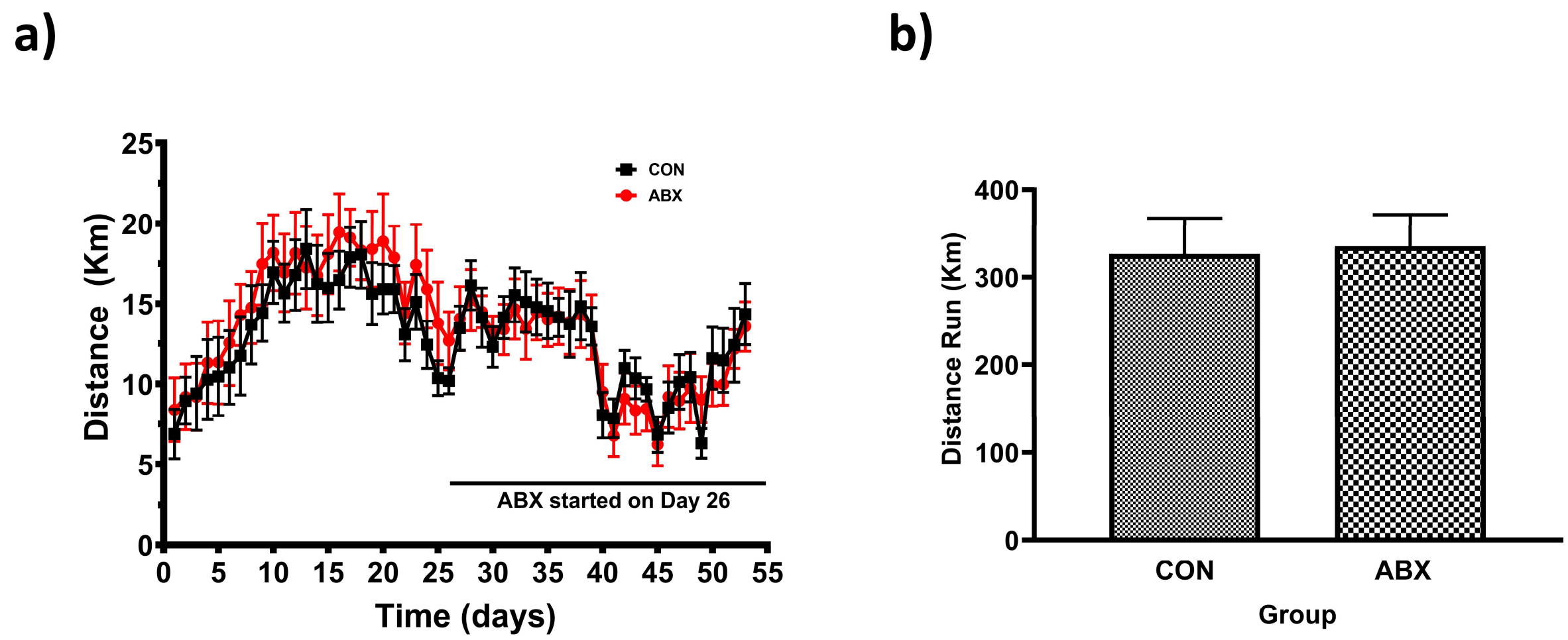

Supplemental Figure 2. Broad spectrum antibiotic treatment does not influence wheel running during the maintenance phase. a) When ABX were administered during 26 days into VWR, they did not affect daily running distance (p=0.78). *N= 5 and 6 per group for CON and VWR, respectively. **b)** Cumulative running distance during the acquisition period (Days 1-20) and during VWR

Figure S3

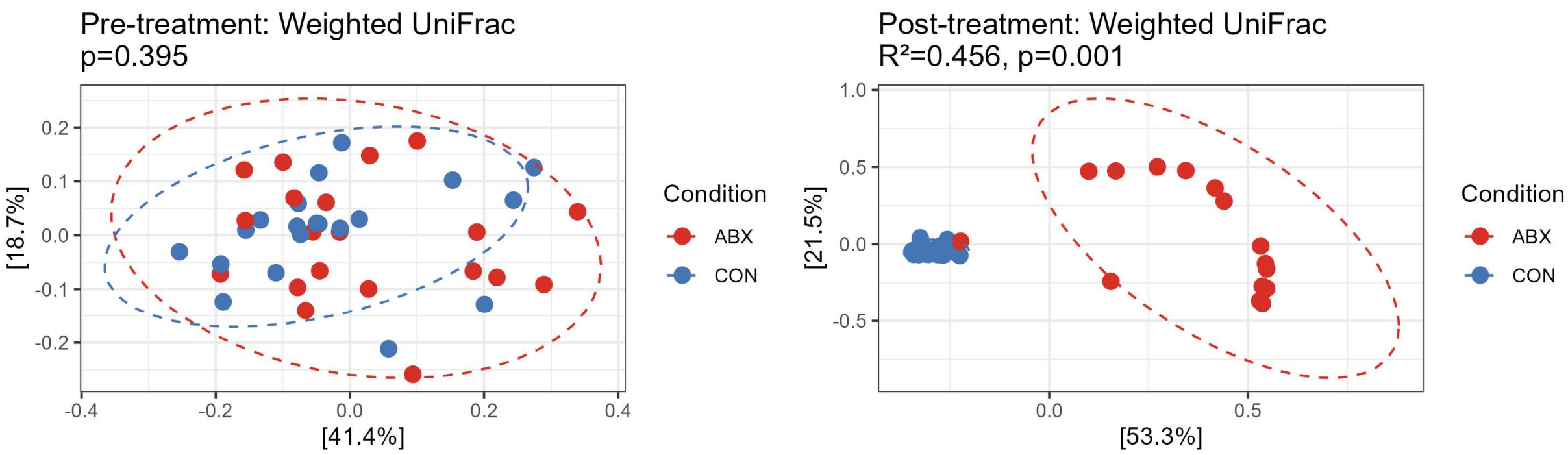

Supplementary Figure S3. Beta diversity of fecal microbial communities before and after antibiotic treatment. Principal coordinates analysis (PCoA) of Weighted UniFrac distances comparing antibiotic-treated (ABX, red) and control (CON, blue) mice at pre-treatment baseline (left) and post-treatment (right). ASVs present in fewer than 20% of samples within each timepoint were excluded prior to analysis. Dashed ellipses represent 95% confidence intervals. Community composition did not differ between groups at baseline (PERMANOVA: $R^2 = 0.02584$, $p = 0.395$), confirming the absence of pre-existing differences between animals subsequently assigned to each condition. Post-treatment, ABX mice exhibited marked separation from controls (PERMANOVA: $R^2 = 0.456$, $p = 0.001$), reflecting antibiotic-induced disruption of the gut microbiome. PERMANOVA was performed with 999 permutations.

Figure S4

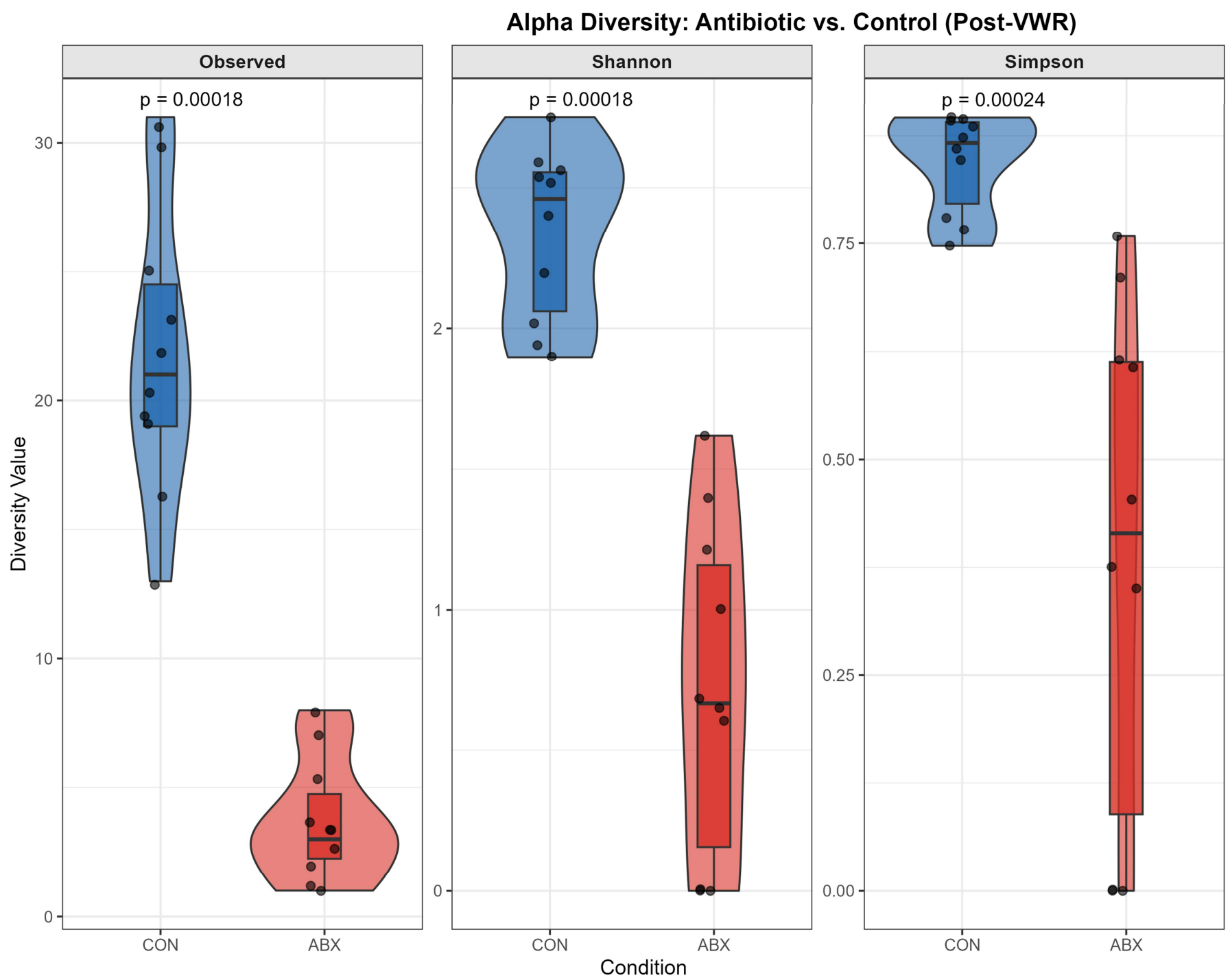

Supplemental Figure S4. Antibiotic Treatment Significantly Reduces Gut Microbiome Alpha Diversity.

Alpha diversity metrics were calculated from 16S rRNA sequencing data after filtering to retain ASVs present in ≥10% of samples. Post-VWR fecal samples from antibiotic-treated (ABX, n=10) and control (CON, n=10) mice were analyzed. Violin plots show the distribution of Observed ASVs, Shannon diversity index, and Simpson diversity index. Boxplots (white boxes) indicate median and interquartile range; individual data points are overlaid. Statistical comparisons performed using Wilcoxon rank-sum test. Antibiotic treatment significantly reduced all diversity metrics: Observed ASVs ($p = 0.00018$), Shannon diversity ($p = 0.00018$), and Simpson diversity ($p = 0.00024$), consistent with broad-spectrum microbiome depletion. P-values displayed above each comparison.

Figure S5

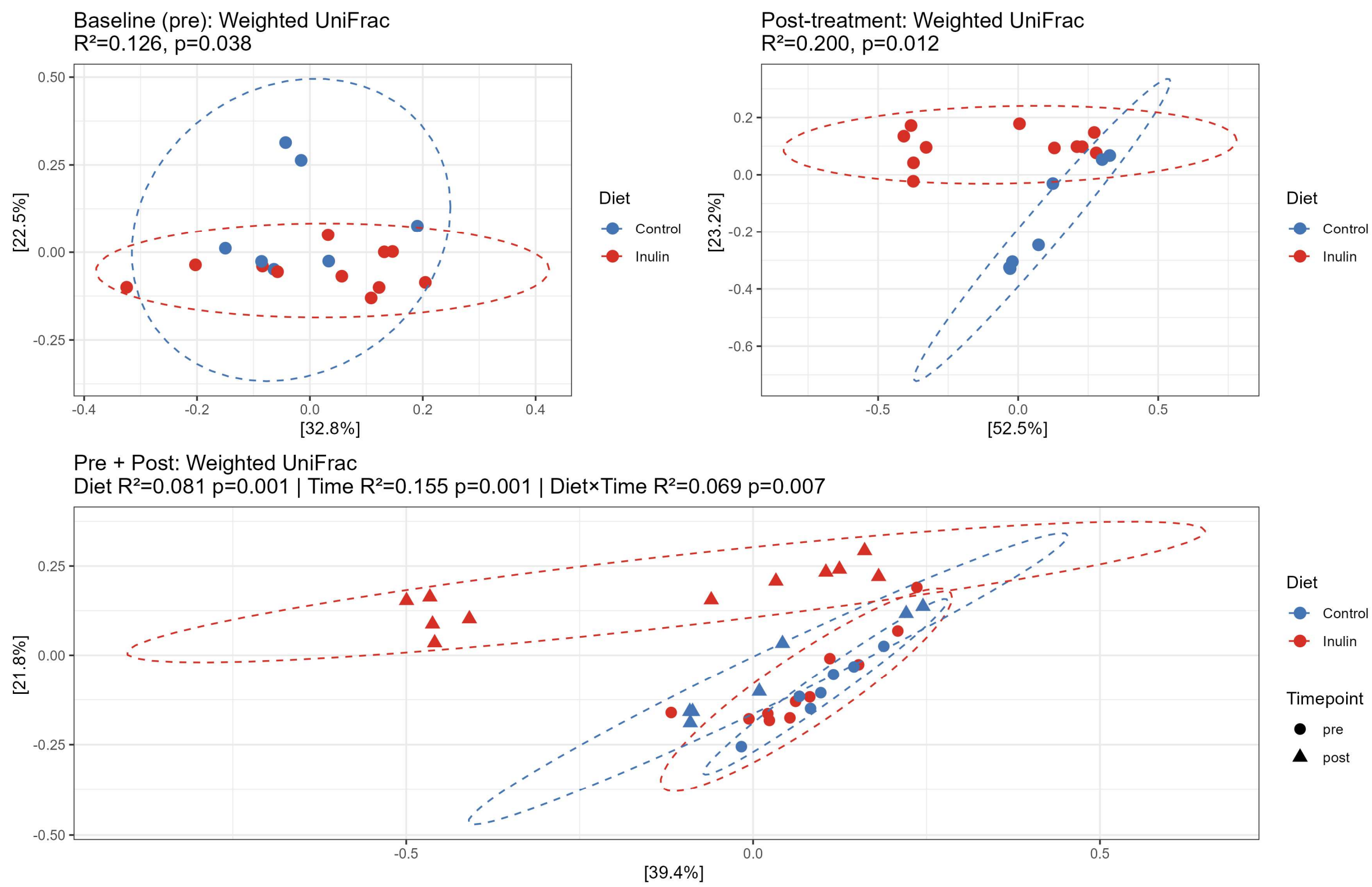

Supplemental Figure S5. Beta diversity of fecal microbial communities in inulin- and control-fed adult mice (Weighted UniFrac).

Principal coordinates analysis (PCoA) of Weighted UniFrac distances in adult mice fed either a control (0% inulin, blue) or inulin-supplemented (2.5%, red) diet. ASVs present with fewer than 5 counts in fewer than 10% of samples were excluded prior to analysis. Dashed ellipses represent 95% confidence intervals. (A) Cross-sectional comparison at the pre-treatment baseline timepoint revealed modest but statistically significant separation between groups (PERMANOVA: $R^2 = 0.126$, $p = 0.038$), indicating some pre-existing differences in community structure prior to dietary intervention. (B) Cross-sectional comparison at the post-treatment timepoint showed greater separation between diet groups (PERMANOVA: $R^2 = 0.200$, $p = 0.012$), with a larger effect size than observed at baseline, suggesting an additional diet-driven divergence in community composition beyond pre-existing differences. (C) Longitudinal analysis including both timepoints (circles = pre, triangles = post) with permutations restricted within individual animals to account for repeated measures. PERMANOVA revealed significant effects of diet ($R^2 = 0.081$, $p = 0.001$), time ($R^2 = 0.155$, $p = 0.001$), and a significant diet × time interaction ($R^2 = 0.069$, $p = 0.007$), indicating that inulin and control groups diverged over the course of the intervention. PERMANOVA was performed with 999 permutations.

Figure S6

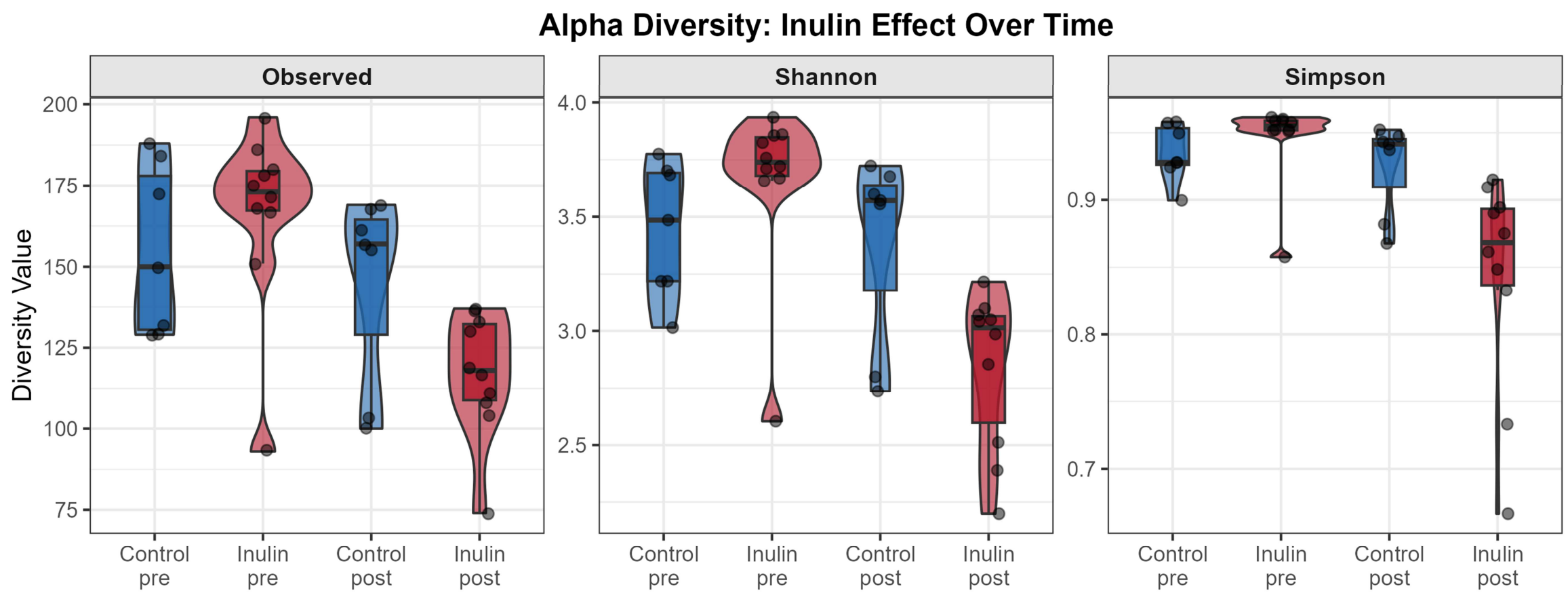

Supplemental Figure S6. Inulin Supplementation Reduces Gut Microbiome Alpha Diversity. Alpha diversity in Control (blue, n=7) and Inulin (red, n=10) groups before (Pre) and after (Post) 8 weeks of dietary intervention, represented by observed ASVs, Shannon Index, and Simpson Index. ASVs were filtered at ≥10% prevalence before diversity calculation. Linear mixed models with Diet × Timepoint interaction revealed significant effects for Shannon diversity ($p = 0.00046$), Simpson diversity ($p = 0.014$), and Observed ASVs ($p = 0.0035$). Inulin treatment reduced all diversity metrics (Shannon: 3.66→2.84; Simpson: 0.946→0.843; Observed: 166→117) while Control remained stable, reflecting taxonomic restructuring toward specialized fiber-degrading taxa with reduced overall diversity.

Figure S7

A

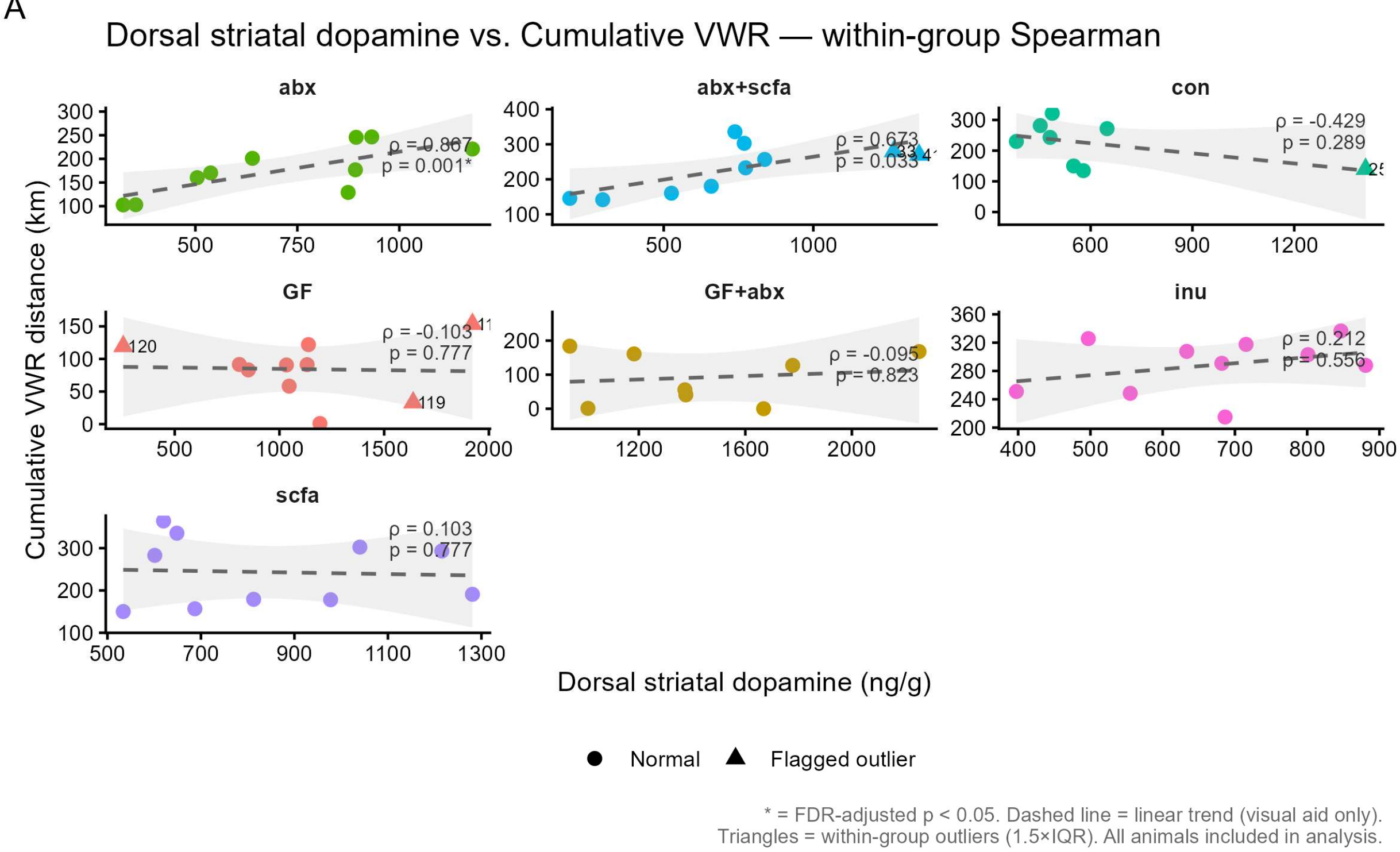

Dorsal striatal dopamine vs. Cumulative VWR — within-group Spearman
abx
abx+scfa
con
GF
GF+abx
inu
scfa
ρ = 0.867
p = 0.001*
ρ = 0.673
p = 0.033
ρ = -0.429
p = 0.289
ρ = -0.103
p = 0.777
ρ = -0.095
p = 0.823
ρ = 0.212
p = 0.556
ρ = 0.103
p = 0.777
Cumulative VWR distance (km)
Dorsal striatal dopamine (ng/g)
Normal
Flagged outlier
* = FDR-adjusted p < 0.05. Dashed line = linear trend (visual aid only).
Triangles = within-group outliers (1.5×IQR). All animals included in analysis.

B

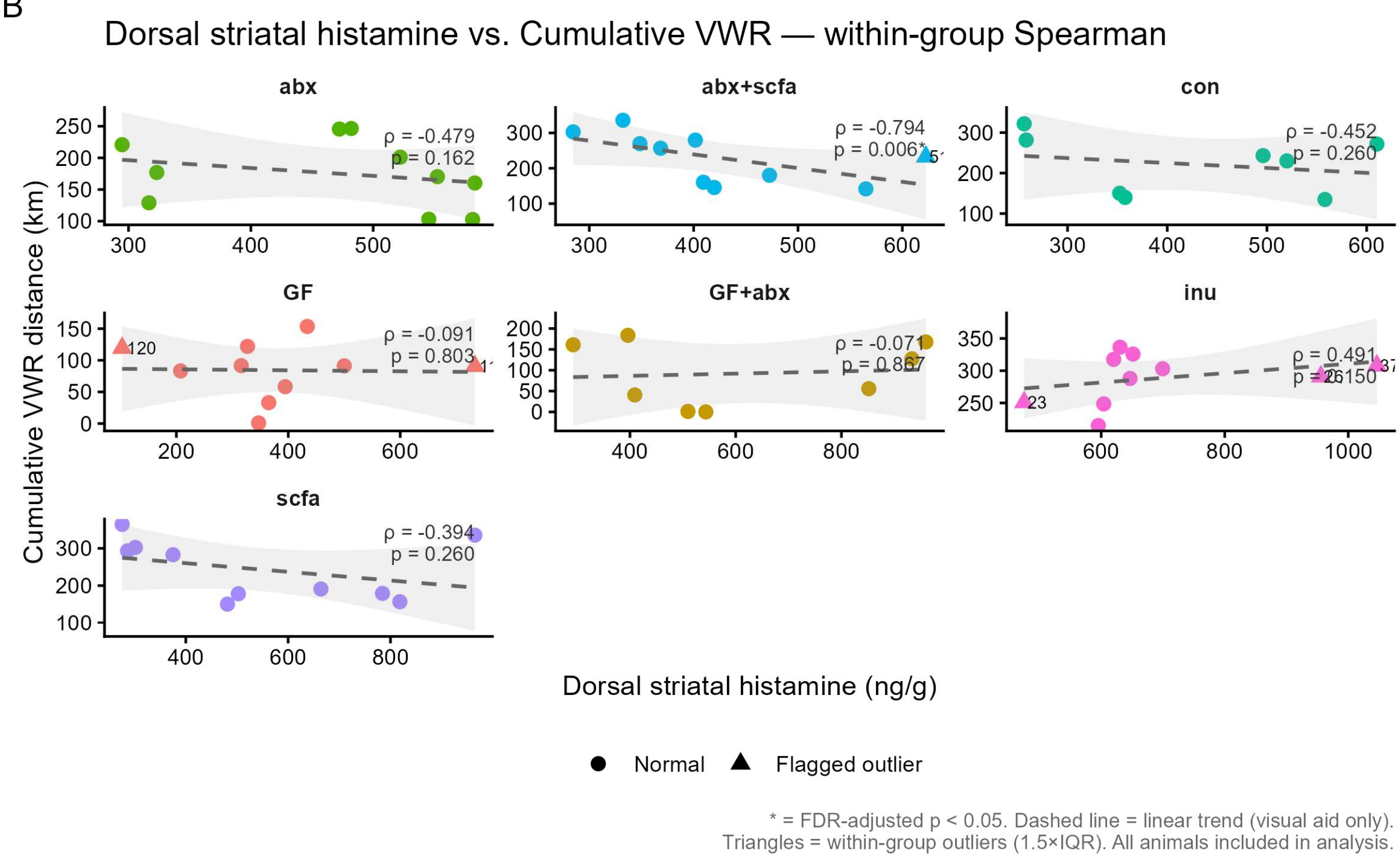

Dorsal striatal histamine vs. Cumulative VWR — within-group Spearman
abx
abx+scfa
con
GF
GF+abx
inu
scfa
ρ = -0.479
p = 0.162
ρ = -0.794
p = 0.006*
ρ = -0.452
p = 0.260
ρ = -0.091
p = 0.803
ρ = -0.071
p = 0.867
ρ = 0.491
p = 0.150
ρ = -0.394
p = 0.260
Cumulative VWR distance (km)
Dorsal striatal histamine (ng/g)
Normal
Flagged outlier
* = FDR-adjusted p < 0.05. Dashed line = linear trend (visual aid only).
Triangles = within-group outliers (1.5×IQR). All animals included in analysis.

Supplementary Figure S7. Within-group Spearman correlations between dorsal striatal neurochemical concentrations and cumulative voluntary wheel running distance.

Each point represents one animal. Correlations were computed within treatment group using Spearman's rank coefficient; p-values were adjusted for multiple comparisons across all group × neurochemical combinations (n = 14 tests) using the Benjamini-Hochberg false discovery rate (BH-FDR) procedure. Spearman ρ, raw p-value, and BH-FDR-adjusted p-value are annotated in the upper right of each panel; asterisk (*) denotes FDR-adjusted $p < 0.05$. Dashed lines and shaded bands indicate linear trends (visual aid only; Spearman correlation is non-parametric). Triangles indicate animals whose neurochemical or VWR value exceeded 1.5 × IQR of the within-group distribution; these animals were retained in all analyses. (A) Dorsal striatal dopamine (ng/mg tissue) versus cumulative VWR distance. (B) Dorsal striatal histamine (ng/g tissue) versus cumulative VWR distance. Groups: ABX, antibiotic-treated; ABX+SCFA, antibiotic-treated with short-chain fatty acid supplementation; CON, conventional control; GF, germ-free; GF+ABX, germ-free antibiotic-treated; SCFA, SCFA supplementation without antibiotics; Inu, inulin diet.
