# Supplementary material for: Fermentation capacity of the gut microbiota influences exercise motivation and neuroendocrine integration
Source: mSystems. 2026 Aug 19;11(9):e00876-26. doi: 10.1128/msystems.00876-26 (PMC13595965; doi:10.1128/msystems.00876-26)
Supplement: Supplementary file 2 [file msystems.00876-26-s0003.docx]

**Supplementary Table S1. Effect sizes and retrospective power for primary outcome (cumulative voluntary wheel running distance)**

| **Cohort** | **Comparison** | **n/group** | **η²p** | **Cohen's f** | **Power (simr, mixed model)¹** | **95% CI (simr)** | **Power (RM-ANOVA, GG-corrected)²** |
| --- | --- | --- | --- | --- | --- | --- | --- |
| ABX (2-arm) | Treatment (ABX vs. CON) | 9 | 0.253 | 0.583 | 0.95 | [0.93, 0.97] | 0.21 |
| ABX × SCFA (2×2) | ABX main effect | ≥14 | 0.121 | 0.370 | 1.00 | [0.99, 1.00] | 0.29 |
| ABX × SCFA (2×2) | ABX × SCFA interaction | ≥14 | 0.033 | 0.186 | 0.93 | [0.91, 0.95] | 0.08 |
| Inulin | Diet main effect | 10 | 0.133 | 0.391 | 1.00 | [0.99, 1.00] | 0.16 |

¹ Power estimated via parametric bootstrap simulation (500 simulations) from the fitted linear mixed-effects model (treatment × day, random intercept per animal), testing whether the full model significantly outperformed a day-only baseline (likelihood ratio test, p<0.05).
² Power estimated via classical repeated-measures ANOVA with Greenhouse-Geisser correction for sphericity (empirical ε≈0.25 across cohorts), shown as a conservative sensitivity check; see Methods for interpretation.

**Supplementary Table S2. Parameters used for Liquid Chromatography/Mass Spectrometry Analysis**

| Q1 | Q2 | Compound | CE |
| --- | --- | --- | --- |
| 104.1 | 45.1 | GABA | 25 |
| 112.1 | 68.1 | Histamine | 28 |
| 138.1 | 77.1 | Tyramine | 37 |
| 147.1 | 88.1 | Acetylcholine | 20 |
| 154.1 | 91.1 | Dopamine | 32 |
| 170.1 | 107.1 | Norepinephrine | 26 |
| 177 | 115.1 | Serotonin | 39 |
| 184.1 | 107.1 | Epinephrine | 30 |
| 198.1 | 152.1 | L-DOPA | 19 |
| 158 | 141 | Dopamine d4 | 35 |
| 181 | 122 | HVA | -31 |
| 167 | 123 | DHPAA | -12 |

**Supplementary Table S3. Within-group Spearman correlations between striatal neurochemical concentrations and cumulative voluntary wheel running distance**

| **Group** | **Neurochemical** | **n** | **Spearman ρ** | **p-value** | **FDR-adjusted p** | **Significant (FDR<0.05)** | **Outliers flagged** |
| --- | --- | --- | --- | --- | --- | --- | --- |
| ABX | Dopamine | 10 | 0.867 | 0.0012 | 0.0164 | Yes | 0 |
| ABX+SCFA | Dopamine | 10 | 0.673 | 0.0330 | 0.1542 | No | 2 |
| CON | Dopamine | 8 | -0.429 | 0.2894 | 0.5065 | No | 1 |
| GF | Dopamine | 10 | -0.103 | 0.7770 | 0.8665 | No | 2 |
| GF+ABX | Dopamine | 8 | -0.095 | 0.8225 | 0.8665 | No | 0 |
| SCFA | Dopamine | 10 | 0.103 | 0.7770 | 0.8665 | No | 0 |
| Inulin | Dopamine | 10 | 0.212 | 0.5563 | 0.8654 | No | 0 |
| ABX | Histamine | 10 | -0.479 | 0.1615 | 0.4523 | No | 0 |
| ABX+SCFA | Histamine | 10 | -0.794 | 0.0061 | 0.0427 | Yes | 1 |
| CON | Histamine | 8 | -0.452 | 0.2604 | 0.5065 | No | 0 |
| GF | Histamine | 10 | -0.091 | 0.8028 | 0.8665 | No | 2 |
| GF+ABX | Histamine | 8 | -0.071 | 0.8665 | 0.8665 | No | 0 |
| SCFA | Histamine | 10 | -0.394 | 0.2600 | 0.5065 | No | 0 |
| Inulin | Histamine | 10 | 0.491 | 0.1497 | 0.4523 | No | 3 |

Cumulative voluntary wheel running distance (summed daily distance over the full VWR period) was correlated with dorsal striatal dopamine and histamine concentrations measured immediately following a controlled post-VWR treadmill exercise bout, within each treatment group separately. Benjamini-Hochberg FDR correction applied across all 14 comparisons. "Outliers flagged" indicates the number of animals per group identified as outliers (1.5×IQR rule, within-group) for the corresponding neurochemical or VWR value; all animals were retained in all analyses regardless of outlier status. Individual flagged animals are labeled in Supplementary Figure S7.
